# Fractional and stochastic modeling of breast cancer progression with real data validation

**DOI:** 10.1371/journal.pone.0313676

**Published:** 2025-01-10

**Authors:** Khaled Aldwoah, Hanen Louati, Nedal Eljaneid, Tariq Aljaaidi, Faez Alqarni, AbdelAziz Elsayed

**Affiliations:** 1 Department of Mathematics, Faculty of Science, Islamic University of Madinah, Madinah, Saudi Arabia; 2 Department of Mathematics, Faculty of Science, Northern Border University, Arar, Saudi Arabia; 3 Department of Mathematics, Faculty of Science, University of Tabuk, Tabuk, Saudi Arabia; 4 Department of Artificial Intelligence, Faculty of Computer Science and Information Technology, Alrazi University, Sana’a, Yemen; 5 Department of General Studies, University of Prince Mugrin (UPM), Madinah, Saudi Arabia; 6 Biology Department, Faculty of Science, Islamic University of Madinah, Madinah, Saudi Arabia; Yarmouk University Hijjawi Faculty for Engineering Technology, JORDAN

## Abstract

This study presents a novel approach to modeling breast cancer dynamics, one of the most significant health threats to women worldwide. Utilizing a piecewise mathematical framework, we incorporate both deterministic and stochastic elements of cancer progression. The model is divided into three distinct phases: (1) initial growth, characterized by a constant-order Caputo proportional operator (CPC), (2) intermediate growth, modeled by a variable-order CPC, and (3) advanced stages, capturing stochastic fluctuations in cancer cell populations using a stochastic operator. Theoretical analysis, employing fixed-point theory for the fractional-order phases and Ito calculus for the stochastic phase, establishes the existence and uniqueness of solutions. A robust numerical scheme, combining the nonstandard finite difference method for fractional models and the Euler-Maruyama method for the stochastic system, enables simulations of breast cancer progression under various scenarios. Critically, the model is validated against real breast cancer data from Saudi Arabia spanning 2004-2016. Numerical simulations accurately capture observed trends, demonstrating the model’s predictive capabilities. Further, we investigate the impact of chemotherapy and its associated cardiotoxicity, illustrating different treatment response scenarios through graphical representations. This piecewise fractional-stochastic model offers a powerful tool for understanding and predicting breast cancer dynamics, potentially informing more effective treatment strategies.

## 1 Introduction

Breast cancer is a complex and diverse illness that originates from the uncontrolled growth of cells in the breast tissue. It is one of the most common cancers among women worldwide [1]. In 2004, the World Health Organization (WHO) ranked breast cancer as the second most common cancer worldwide. As per WHO statistics, breast cancer affects 8 to 9 percent of women globally. Global health researchers remain uncertain about the precise causes of breast cancer [2]. In 2020, breast cancer impacted 2.3 million women worldwide and led to 685,000 deaths [3]. In 2022, breast cancer caused 670,000 deaths globally [4]. Certain factors increase the risk of breast cancer such as tobacco use, age, family history and genetics, hormonal factors, obesity, alcohol consumption, and radiation exposure. Common symptoms of breast cancer include a lump in the breast, changes in breast size and shape, skin changes on the breast, nipple discharge, inverted nipple, and redness of the breast skin. For prediction and forecasting of breast cancer, mathematicians develop several mathematical models in the literature [5–7].

Over time, there have been significant advancements in understanding the diagnosis, development, and cure of breast cancer. Despite significant progress in understanding breast cancer over the past 50 years, it remains a big public health concern worldwide. Breast cancer is the most frequently occurring invasive cancer affecting women worldwide [8]. Breast cancer ranked as the most commonly diagnosed cancer in Saudi Arabia in 2018, following leukemia as the second-leading cause of death [9]. While Saudi Arabia has historically had a lower incidence of breast cancer compared to different Western nations, increasing evidence suggests a rapid rise in the incidence rates. Investigating breast cancer features, fashions, age distributions, and regional differences is considered a leading research area in developing countries like Saudi Arabia, where over 70 percent of women are below 39 years old.

In the paper [10], the authors have formulated the mathematical model for breast cancer. As per the medical records, admitted patients are categorized into subgroups ranging from stage 1 to stage 4. The population is categorized into five subclasses:

$$\begin{array}{ccc} \frac{d\text{X}}{dt} & = & \omega_{1}-\left(\gamma_{1}+\mu_{2}\right)\text{X},\\ \frac{d\text{B}}{dt} & = & \omega_{2}+\mu_{2}\text{X}+\alpha_{1}\text{R}-\left(\gamma_{2}+\mu_{1}+\phi_{1}+\sigma_{1}\right)\text{B},\\ \frac{d\text{C}}{dt} & = & \omega_{3}+\mu_{1}\text{B}+\alpha_{2}\text{R}-\left(\gamma_{3}+\phi_{2}+\sigma_{2}\right)\text{C},\\ \frac{d\text{R}}{dt} & = & \gamma_{1}\text{X}+\gamma_{2}\text{B}+\gamma_{3}\text{C}-\left(\alpha_{1}+\alpha_{2}+\phi_{3}\right)\text{R},\\ \frac{d\text{E}}{dt} & = & \phi_{3}\text{R}+\phi_{2}\text{C}+\phi_{1}\text{B}-\sigma_{3}\text{E}, \end{array}$$ (1)

with initial values **X** > 0 **B** ≥ 0, **C** ≥ 0, **R** ≥ 0 and **E** ≥ 0. The class **X** denotes the patients hospitalized due to the impact of cancer who have stage 1, the class **B** describes individuals identified as patients with stage 3**X** and stage 3**B**, the class **C** contains the people who undergo testing and are diagnosed as cancer patients, the class **R** denotes individuals who are in disease-free states following chemotherapy treatment and the class **E** represents cancer patients experiencing cardiotoxic effects.

In recent years, fractional calculus (FC) has received tremendous attention of researchers due to several applications in different fields of science and engineering [11]. FC has been used in the analysis of physical system which occurs in mathematical biology [12, 13], physics and engineering [14] and many more [15, 16]. Scientists have been actively exploring novel fractional operators to tackle challenges related to memory, locality, and singularity. The Caputo fractional derivative (CFD) has proven to be a highly valuable operator for representing non-local behaviors through fractional differential equations. In reference [17], the authors proposed a novel hybrid fractional operator by innovatively combining the concepts of the CFD and the proportional derivative (CPC operator). This hybrid fractional operator is designed as a linear combination of the CFD and the Riemann–Liouville integral of non-integer order. Only few works have been published using CPC operator [18, 19].

Disease often exhibit random behaviour due to various factors such as genetic mutations, environmental influences, and stochastic processes within biological systems, Stochastic differential equations (SDEs) allow for the inclusion of stochastic components, making them suitable for capturing the inherent uncertainty in disease dynamics. Several researchers have used SDEs for modelling various kinds of diseases such as COVID-19 [20], HIV [21] and some others [22, 23].

Recently, Abdon and Seda [24] introduced a hybrid non-local operator based on a piece-wise (PW) function approach. This PW operator involves dividing the interval into two or more sub-intervals, and the authors have proposed various types of PW operators by combining different operators within these sub intervals. The PW operator is specially used for analysis of crossover behaviour in the dynamics of a physical system. Some disease models have been investigated using PW operators [25–27]. Motivated by works on PW operator, we aim to study the considered breast cancer model under PW. Consider the considered model under PW operator as:

$$\begin{array}{c} \left\{ \begin{array}{c} {\text{D}}_{t}^{PW}\text{X}\left(t\right)=\omega_{1}-\left(\gamma_{1}+\mu_{2}\right)\text{X},\\ {\text{D}}_{t}^{PW}\text{B}\left(t\right)=\omega_{2}+\mu_{2}\text{X}+\alpha_{1}\text{R}-\left(\gamma_{2}+\mu_{1}+\phi_{1}+\sigma_{1}\right)\text{B},\\ {\text{D}}_{t}^{PW}\text{C}\left(t\right)=\omega_{3}+\mu_{1}\text{B}+\alpha_{2}\text{R}-\left(\gamma_{3}+\phi_{2}+\sigma_{2}\right)\text{C},\\ {\text{D}}_{t}^{PW}\text{R}\left(t\right)=\gamma_{1}\text{X}+\gamma_{2}\text{B}+\gamma_{3}\text{C}-\left(\alpha_{1}+\alpha_{2}+\phi_{3}\right)\text{R},\\ {\text{D}}_{t}^{PW}\text{E}\left(t\right)=\phi_{3}\text{R}+\phi_{2}\text{C}+\phi_{1}\text{B}-\sigma_{3}\text{E}, \end{array}\right. \end{array}$$ (2)

where ${\text{D}}_{t}^{PW}$ represents a PW differential operator, which will be defined later section for three subintervals. In this paper, we utilize the PW operator in a unique manner, employing the CPC operator in the first two sub-intervals with constant and variable order, respectively. The stochastic operator in the other sub interval, to analyze random behaviour the proposed model 1. This type of pattern is used only for COVID-19 model [28]. The PW formulation of the model is expressed as follows:

{1λ1-θD0CPCtθX(t)=ω1-(γ1+μ2)X,1λ1-θD0CPCtθB(t)=ω2+μ2X+α1R-(γ2+μ1+ϕ1+σ1)B,1λ1-θD0CPCtθC(t)=ω3+μ1B+α2R-(γ3+ϕ2+σ2)C,0<t≤T1,0<θ<11λ1-θD0CPCtθR(t)=γ1X+γ2B+γ3C-(α1+α2+ϕ3)R,1λ1-θD0CPCtθE(t)=ϕ3R+ϕ2C+ϕ1B-σ3E, (3)

with initial values is

$$\begin{array}{cc} & \text{X}\left(0\right)={\text{X}}_{0}\geq 0,\text{B}\left(0\right)={\text{B}}_{0}\geq 0,\text{C}\left(0\right)={\text{C}}_{0}\geq 0,\text{R}\left(0\right)={\text{R}}_{0}\geq 0,\text{E}\left(0\right)={\text{E}}_{0}\geq 0. \end{array}$$ (4)

For variable order, we can write the above model 3 as given below:

{1λ1-θ(t)D0CPCtθ(t)X(t)=ω1-(γ1+μ2)X,1λ1-θ(t)D0CPCtθ(t)B(t)=ω2+μ2X+α1R-(γ2+μ1+ϕ1+σ1)B,1λ1-θ(t)D0CPCtθ(t)C(t)=ω3+μ1B+α2R-(γ3+ϕ2+σ2)C,0<t≤T1,0<θ<1,1λ1-θ(t)D0CPCtθ(t)R(t)=γ1X+γ2B+γ3C-(α1+α2+ϕ3)R,1λ1-θ(t)D0CPCtθ(t)E(t)=ϕ3R+ϕ2C+ϕ1B-σ3E, (5)

with initial values is

$$\begin{array}{cc} & \text{X}\left({\text{T}}_{1}\right)={\text{X}}_{1}\geq 0,\text{B}\left({\text{T}}_{1}\right)={\text{B}}_{1}\geq 0,\text{C}\left({\text{T}}_{1}\right)={\text{C}}_{1}\geq 0,\text{R}\left({\text{T}}_{1}\right)={\text{R}}_{1}\geq 0,\\ & \text{E}\left({\text{T}}_{1}\right)={\text{E}}_{1}\geq 0. \end{array}$$

The stochastic version of system 2 is expressed by:

$$\begin{array}{c} \left\{ \begin{array}{c} d\text{X}\left(t\right)=[\omega_{1}-\left(\gamma_{1}+\mu_{2}\right)\text{X}]dt+\varsigma_{1}\text{X}\left(t\right)dM_{1}^{{\text{H}}^{⋆}}\left(t\right),\\ d\text{B}\left(t\right)=[\omega_{2}+\mu_{2}\text{X}+\alpha_{1}\text{R}-\left(\gamma_{2}+\mu_{1}+\phi_{1}+\sigma_{1}\right)\text{B}]dt+\varsigma_{2}\text{B}\left(t\right)dM_{2}^{{\text{H}}^{⋆}},\\ d\text{C}\left(t\right)=[\omega_{3}+\mu_{1}\text{B}+\alpha_{2}\text{R}-\left(\gamma_{3}+\phi_{2}+\sigma_{2}\right)\text{C}]dt+\varsigma_{3}\text{E}\left(t\right)dM_{3}^{{\text{H}}^{⋆}},\\ d\text{R}\left(t\right)=[\gamma_{1}\text{X}+\gamma_{2}\text{B}+\gamma_{3}\text{C}-\left(\alpha_{1}+\alpha_{2}+\phi_{3}\right)\text{R}]dt\\ +\varsigma_{4}\text{B}\left(t\right)dM_{4}^{{\text{H}}^{⋆}},\;\;\;\;\;\;\;\;\;\;\;\;\;\;\;\;\;\;\;\;\;{\text{T}}_{2}<t\leq {\text{T}}_{f}\\ d\text{E}\left(t\right)=[\phi_{3}\text{R}+\phi_{2}\text{C}+\phi_{1}\text{B}-\sigma_{3}\text{E}]dt+\varsigma_{5}\text{Z}\left(t\right)dM_{5}^{{\text{H}}^{⋆}}, \end{array}\right. \end{array}$$ (6)

with initial values is

$$\begin{array}{cc} & \text{X}\left({\text{T}}_{2}\right)={\text{X}}_{2}\geq 0,\text{B}\left({\text{T}}_{2}\right)={\text{B}}_{2}\geq 0,\text{C}\left({\text{T}}_{2}\right)={\text{C}}_{2}\geq 0,\text{R}\left({\text{T}}_{2}\right)={\text{R}}_{2}\geq 0,\\ & \text{E}\left({\text{T}}_{2}\right)={\text{E}}_{2}\geq 0,, \end{array}$$

in the above model $M_{i}\left(t\right)\;\;for\;\;i=1,2,\ldots ,5$ were the densities and $\varsigma_{i}\;\;for\;\;i=1,2,\ldots ,5$ are the noise intensities, while **H**^⋆^ is the Hurst index.

## 2 Basic concepts

Presently, we define some basic concepts which will be use in our proposed work.

**Definition 2.1** [11] *Suppose* g(t) *is a function which is continuous given by* Ψ = [*ω*, *ν*], −∞ < *ω* < *ν* < +∞, *θ* ∈ **C**, **R**(*θ*) > 0. *The Riemann-Liouville derivative is defined under, both left and right for order θ*:

Dωtθg(t)=1Γ(n-θ)(ddt)n∫ωtg(s)(t-s)1-n+θds,t>ω,Dtνθg(t)=1Γ(n-θ)(-ddt)n∫tνg(s)(s-t)1-n+θds,t<ν, (7)

*where* n=[R(θ)]+1.

**Definition 2.2** [11] *Suppose* g(t) *is a function which is continuous given by* Ψ = [*ω*, *ν*], −∞ < *ω* < *ν* < +∞, *θ* ∈ **C**, **R**(*θ*) > 0. *The Riemann-Liouville integrals are defined under, both left and right for order θ*:

IωRLtθg(t)=ωDt-θg(t)=1Γ(θ)[∫ωtg(s)(t-s)θ-1ds],t>ω,ItRLνθg(t)=tDν-θg(t)=1Γ(θ)[∫ωtg(s)(t-s)θ-1ds],t<ν, (8)

*where* 0 < *θ* < 1.

**Definition 2.3** [11] *Let* g(t) *be function in* **C**, *then the Caputo derivative for order θ is defined below*,

DωCtθg(t)=1Γ(n-β)∫ωtgn(s)(t-ζ)1-n+θds,t>ω,DtCνθg(t)=(-1)nΓ(n-β)∫tνgn(s)(s-t)1-n+θds,t<ν, (9)

*where* n=[R(θ)]+1.

**Definition 2.4** [17] *Let the Caputo proportional Fractional Hybrid operator is presented by (CP) then (CP) is defined as*;

D0CPtθf(t)=(∫0t(f(s)Z1(s,θ)+f′(s)Z0(sθ))(t-s)-θds)1Γ(1-θ),=(Z1(t,θ)f(t)+Z0(t,θ)f′(t))(t-θΓ(1-θ)), (10)

*where* ${\text{Z}}_{0}\left(\theta ,t\right)=\theta t^{1-\theta },\;\;{\text{Z}}_{1}\left(\theta ,t\right)=\left(1-\theta \right)t^{\theta }$, *and* 0 < *θ* < 1.

**Definition 2.5** [17] *Let the Fractional hybrid operator along with Caputo proportional Constant is presented by (CPC), and is defined as*;

D0CPCtθf(t)=(∫0t(t-s)-θ(f(s)Z1(θ)+f′(s)Z0(θ))ds)1Γ(1-θ),=(Z1(θ)0RLIt1-θf(t)+Z0(θ)0CDtθf(t)), (11)

*where* **Z**_0_(*θ*) = *θ* **Q**^1−*θ*^, **Z**_1_(*θ*) = (1 − *θ*)**Q**^*θ*^, *are kernels and* **Q** *is constant also* 0 < *θ* < 1.

**Definition 2.6** [17] *Fractional Caputo proportional operator (CP) for variable order as defined as*:

D0CPtθ(t)f(t)=∫0t(Γ(1-θ(t)))-1(t-s)-θ(t)(f′(s)Z0(s,θ(t))+f(s)Z1(s,θ(t)))ds=(Γ(1-θ(t))-1tθ(t))(f′(t)Z0(t,θ(t))+f(t)Z1(t,θ(t))), (12)

*where* ${\text{Z}}_{1}\left(\theta \left(t\right),t\right)=\left(-\theta \left(t\right)+1\right)t^{\theta (t)},{\text{Z}}_{0}\left(\theta \left(t\right),t\right)=t^{\left(1-\theta (t)\right)}\theta \left(t\right)$, *and* 1>θ(t)>0. *Consequently, the variable order CPC can be written as*:

D0CPCtθ(t)f(t)=(∫0t(t-s)-θ(t)1Γ(1-θ(t))(f(s)Z1(θ(t))+f′(s)Z0(θ(t)))ds)=Z1(θ(t))0RLIt1-θ(t)f(t)+Z0(θ(t))0CDtθ(t)f(t), (13)

*where* ${\text{Z}}_{0}\left(\theta \left(t\right)\right)=\theta \left(t\right){\text{Q}}^{\left(1-\theta (t)\right)},\;\;{\text{Z}}_{1}\left(\theta \left(t\right)\right)=\left(1-\theta \left(t\right)\right){\text{Q}}^{\theta (t)}$, *and* **Q** *is constant also* 1>θ(t)>0.

*The inverse operator of the above is given below*:

D0CPCtθ(t)f(t)=(∫0texp[Z1(θ(t))Z0(θ(t))(t-s)]0RLDt(1-θt)f(s)ds)1Z0(θ(t)). (14)

## 3 Theoretical and numerical analysis

We prove some results for the existence and uniqueness of the proposed model 2. We also present a numerical scheme for this model (2).

### 3.1 The existence and uniqueness of model 3 and 5

In this section, we elaborate on the existence and uniqueness of considered models 3 and 5. So we will prove the perovs theorem discussed in [29]. To prove this theorem first we will discuss some basic concepts concerning this theorem.

**Definition 3.1** *Let* **K** *be a vector space with field* **V**, *and it be* **C** *or* **R**. *In this context, someone defined a function (generalized norm) on* **K** *as*:

$$\begin{array}{c} {\left||.|\right|}_{M}=\text{K}\to {\left[0,+\infty \right)}^{\text{n}}\\ \Phi \to {\left||\Phi |\right|}_{M}=(\begin{array}{c} {\left||\Phi |\right|}_{1}\\ \vdots \\ {\left||\Phi |\right|}_{\text{n}} \end{array}), \end{array}$$

having the following properties:

(i) For all Φ ∈ **K**; if ${\left||\Phi |\right|}_{M}=0_{{\text{R}}_{+}^{\text{n}}}$, then Φ = 0_**K**_.

(ii) ||b||_*M*_ = |*b*|||Φ||_*M*_ ∀ Φ ∈ **K** and b ∈ **V**, and

(iii) ||Φ + λ||_*M*_ ≤ ||Φ||_*M*_ + ||λ||_*M*_ for all Φ, λ ∈ **K**.

In the above the generalized norm space is (**K**, ||.||_*M*_). Also if the given metric valued space is complete, then the space (**K**, ||.||_*M*_) will form generalized Banach space, having the property *ω*_*M*_(Φ_1_, Φ_2_) = ||Φ_1_ − Φ_2_||_*G*_.

**Definition 3.2** *Suppose* (**K**, λ_*M*_) *be a generalized metric space and let* **P** *be a mapping from* **K** → **K**, *then the operator as known as a* Λ *contraction along with matrix from* **G**_n×n_(**R**_+_) *which goes to* 0_n_, *suggested that*, ∀ *ω*, *μ* ∈ **K**, *the following holds*:

$$\begin{array}{c} \lambda_{M}(\text{P}(\omega ),\text{P}(\mu ))\leq \Lambda \lambda_{M}\left(\omega ,\mu \right). \end{array}$$

*The result given below is the extension of Banach contraction principle*.

**Theorem 1** [29] *Suppose* **K** *is a full generalized metric space also* **P**: **K** → **K** *is a M-contraction operator, then*, **P** *will have a single fixed point belong to* **K**. *Further, the models* 3 *and* 5 *can be written in the following form*,

{DCPCtθY(t)=σ1-θZ(Y(t)),Y(0)=Y00<t<T1<∞, (15)

*in the above equation*, Y(t) *is vector which is equal to* $\text{Y}\left(t\right)={\left({\text{X}}_{0},{\text{B}}_{0},{\text{C}}_{0},{\text{R}}_{0},{\text{E}}_{0}\right)}^{t}$ *also* **Z** *operator is given below*,

$$\begin{array}{c} \text{Z}\left(\text{Y}\right)=[\begin{array}{c} {\text{Z}}_{1}\left(\text{Y}\right)\\ {\text{Z}}_{2}\left(\text{Y}\right)\\ {\text{Z}}_{3}\left(\text{Y}\right)\\ {\text{Z}}_{4}\left(\text{Y}\right)\\ {\text{Z}}_{5}\left(\text{Y}\right) \end{array}]=[\begin{array}{cc} & \omega_{1}-\left(\gamma_{1}+\mu_{2}\right)\text{X}\\ & \omega_{2}+\mu_{2}\text{X}+\alpha_{1}\text{R}-\left(\gamma_{2}+\mu_{1}+\phi_{1}+\sigma_{1}\right)\text{B}\\ & \omega_{3}+\mu_{1}\text{B}+\alpha_{2}\text{R}-\left(\gamma_{3}+\phi_{2}+\sigma_{2}\right)\text{C}\\ & \gamma_{1}\text{X}+\gamma_{2}\text{B}+\gamma_{3}\text{C}-\left(\alpha_{1}+\alpha_{2}+\phi_{3}\right)\text{R}\\ & \phi_{3}\text{R}+\phi_{2}\text{C}+\phi_{1}\text{B}-\sigma_{3}\text{E} \end{array}]. \end{array}$$ (16)

*Let* $\text{K}=\Pi_{i}^{5}\text{C}\left(\left[0,t\right],\text{R}\right)$ *be a generalized Banach space if we define it along with the generalized norm given below*:

$$\begin{array}{c} {\left||.|\right|}_{M}:\text{K}\to {\text{R}}_{+}^{5} \end{array}$$

$$\begin{array}{c} \text{Y}\to {\left||\text{Y}|\right|}_{M}=[\begin{array}{c} {\left||\text{X}|\right|}_{\infty }\\ {\left||\text{B}|\right|}_{\infty }\\ {\left||\text{C}|\right|}_{\infty }\\ {\left||\text{R}|\right|}_{\infty }\\ {\left||\text{E}|\right|}_{\infty } \end{array}]. \end{array}$$ (17)

*Now we have to prove that the models* 3 *and* 5 *possess unique solutions, so we are shifting them into fixed point problems having the operator* **Q**, *which is defined as* $\text{Q}:\Pi_{i=1}^{5}\text{C}\left(\left[0,t\right],\text{R}\right)\to \Pi_{i=1}^{5}\text{C}\left(\left[0,t\right],\text{R}\right)$,

Q(Y(t))=Y(0)+σ1-θV0(θ)∫0texp(-V1(θ)V0(θ)(t)(t-s))D0RLt1−θZ(Y(s))ds. (18)

*Further, we need to prove the following lemma*.

**Lemma 3.1** *Let* Λ *is a vector belongs to* **R**^5^ *and fulfill the following conditions*:

$$\begin{array}{c} \Lambda =[\begin{array}{c} \Lambda_{1}\\ \Lambda_{2}\\ \Lambda_{3}\\ \Lambda_{4}\\ \Lambda_{5} \end{array}]\geq \frac{\lambda^{1-\theta }\Psi_{\left(\theta \right)}^{\text{max}}\Phi_{\left(\theta \right)}^{\text{max}}}{\Gamma \left(\theta -1\right)|{\text{V}}_{0}\left(\theta \right)|}[\begin{array}{cc} & |\omega_{1}-\left(\gamma_{1}+\mu_{2}\right)\Lambda_{1}|\\ & |\omega_{2}+\mu_{2}\Lambda_{1}+\alpha_{1}\Lambda_{4}-\left(\gamma_{2}+\mu_{1}+\phi_{1}+\sigma_{1}\right)\Lambda_{2}|\\ & |\omega_{3}+\mu_{1}\Lambda_{2}+\alpha_{2}\Lambda_{4}-\left(\gamma_{3}+\phi_{2}+\sigma_{2}\right)\Lambda_{3}|\\ & |\gamma_{1}\Lambda_{1}+\gamma_{2}\Lambda_{2}+\gamma_{3}\Lambda_{3}-\left(\alpha_{1}+\alpha_{2}+\phi_{3}\right)\Lambda_{4}|\\ & |\phi_{3}\Lambda_{4}+\phi_{2}\Lambda_{3}+\phi_{1}\Lambda_{2}-\sigma_{3}\Lambda_{5}| \end{array}]. \end{array}$$ (19)

*So*, **Z** *maps* **U**(**Y**_0_, Λ) ⊂ **K** *into itself, where* **U**(**Y**_0_, Λ) *is a generalized ball*. *Further we prove that* **Z** *is* **G**-*Lipschitz*.

**Proof 3.1** *Suppose* **Y** ∈ **U**(**Y**_0_, Λ), *then*

||R(Y)-Y0||M=||λ1-θV0(θ)∫t0exp(-V0(θ)V0(θ)(t-s))D0RLt1−θZ(Y(s))ds||M≤λ1-θV0(θ)∫t0exp(-V0(θ)V0(θ)(t-s))||D0RLt1−θZ(Y(s))||Mds≤λ1-θΨ(θ)maxΓ(θ-1)V0(θ)||∫0t(t-s)θ-2Z(Y(t))ds||M≤λ1-θΨ(θ)maxΦ(θ)maxΓ(θ-1)|V0(θ)|CZ

$$\begin{array}{c} {\text{C}}_{\text{Z}}=\underset{\text{Y}\in \text{U}({\text{Y}}_{0},\Lambda )}{\text{sup}}||\text{Z}(\text{Y})|{|}_{M}\leq \frac{\lambda^{1-\theta }\Psi_{\left(\theta \right)}^{\text{max}}\Phi_{\left(\theta \right)}^{\text{max}}}{\Gamma \left(\theta -1\right)|{\text{V}}_{0}\left(\theta \right)|}[\begin{array}{cc} & |\omega_{1}-\left(\gamma_{1}+\mu_{2}\right)\Lambda_{1}|\\ & |\omega_{2}+\mu_{2}\Lambda_{1}+\alpha_{1}\Lambda_{4}-\left(\gamma_{2}+\mu_{1}+\phi_{1}+\sigma_{1}\right)\Lambda_{2}|\\ & |\omega_{3}+\mu_{1}\Lambda_{2}+\alpha_{2}\Lambda_{4}-\left(\gamma_{3}+\phi_{2}+\sigma_{2}\right)\Lambda_{3}|\\ & |\gamma_{1}\Lambda_{1}+\gamma_{2}\Lambda_{2}+\gamma_{3}\Lambda_{3}-\left(\alpha_{1}+\alpha_{2}+\phi_{3}\right)\Lambda_{4}|\\ & |\phi_{3}\Lambda_{4}+\phi_{2}\Lambda_{3}+\phi_{1}\Lambda_{2}-\sigma_{3}\Lambda_{5}| \end{array}]. \end{array}$$ (20)

*Hence the proof*.

**Lemma 3.2** *The operator defined in theorem 1* **Z** *is M lipschitz i.e* $\Theta \in N_{6}\left({\text{R}}_{+}\right)$ *satisfying the following condition*:

$$\begin{array}{c} ||\text{Z}\left(\text{Y}\right)-\text{Z}\left(\bar{\text{Y}}\right){\left|\right|}_{M}\leq \Theta ||\text{Y}-\bar{\text{Y}}{\left|\right|}_{M}, \end{array}$$

*where* Θ *is a square matrix*.

**Proof 3.2** *Suppose* $\text{Y}=(\text{X},\text{B},\text{C},\text{R},\text{E}),\bar{\text{Y}}=(\bar{\text{X}},\bar{\text{B}},\bar{\text{C}},\bar{\text{R}},\bar{\text{E}})\in \bar{\text{U}}\left({\text{Y}}_{0},\Lambda \right)$ *and we get*,

$$\begin{array}{ccc} \left|{\text{Z}}_{1}\left(\text{Y}\right)-{\text{Z}}_{1}\left(\bar{\text{Y}}\right)\right| & = & \left|\omega_{1}-\left(\gamma_{1}+\mu_{2}\right)\text{X}-\omega_{1}+\left(\gamma_{1}+\mu_{2}\right)\bar{\text{X}}\right|\\ & \leq & \left(\gamma_{1}+\mu_{2}\right)\left|\right|\bar{\text{X}}-\text{X}\left|\right|. \end{array}$$

*Similarly*,

$$\begin{array}{ccc} \left|\right|{\text{Z}}_{2}\left(\text{Y}\right)-{\text{Z}}_{2}\left(\bar{\text{Y}}\right){\left|\right|}_{\infty } & = & \left|\right|\omega_{2}+\mu_{2}\text{X}+\alpha_{1}\text{R}-\left(\gamma_{2}+\mu_{1}+\phi_{1}+\sigma_{1}\right)\text{B}-\omega_{2}-\mu_{2}\bar{\text{X}}-\alpha_{1}\bar{\text{R}}+\left(\gamma_{2}+\mu_{1}+\phi_{1}+\sigma_{1}\right)\bar{\text{B}}\left|\right|\\ & \leq & \left(\mu_{2}\right)||\text{X}-\bar{\text{X}}{\left|\right|}_{\infty }+\left(\alpha_{1}\right)||\text{R}-\bar{\text{R}}{\left|\right|}_{\infty }+\left(\gamma_{2}+\mu_{1}+\phi_{1}+\sigma 1\right)\left|\right|\bar{\text{B}}-\text{B}{\left|\right|}_{\infty }, \end{array}$$

$$\begin{array}{ccc} \left|\right|{\text{Z}}_{3}\left(\text{Y}\right)-{\text{Z}}_{3}\left(\bar{\text{Y}}\right){\left|\right|}_{\infty } & = & \left|\right|\omega_{3}+\mu_{1}\text{B}+\alpha_{2}\text{R}-\left(\gamma_{3}+\phi_{2}+\sigma_{2}\right)\text{C}-\omega_{3}-\mu_{1}\bar{\text{B}}-\alpha_{2}\bar{\text{R}}+\left(\gamma_{3}+\phi_{2}+\sigma_{2}\right)\bar{\text{C}}\left|\right|\\ & \leq & \mu_{1}||\text{B}-\bar{\text{B}}{\left|\right|}_{\infty }+\alpha_{2}||\text{R}-\bar{\text{R}}{\left|\right|}_{\infty }+\left(\gamma_{3}+\phi_{2}+\sigma 2\right)\left|\right|\bar{\text{C}}-\text{C}{\left|\right|}_{\infty }, \end{array}$$

$$\begin{array}{ccc} \left|\right|{\text{Z}}_{4}\left(\text{Y}\right)-{\text{Z}}_{4}\left(\bar{\text{Y}}\right){\left|\right|}_{\infty } & = & \left|\right|\gamma_{1}\text{X}+\gamma_{2}\text{B}+\gamma_{3}\text{C}-\left(\alpha_{1}+\alpha_{2}+\sigma_{3}\right)\text{R}-\gamma_{1}\bar{\text{X}}-\gamma_{1}\bar{\text{B}}-\gamma_{3}\bar{\text{C}}+\left(\alpha_{1}+\alpha_{2}+\sigma_{3}\right)\bar{\text{R}}\left|\right|\\ & \leq & \gamma_{1}||\text{X}-\bar{\text{X}}{\left|\right|}_{\infty }+\gamma_{2}||\text{B}-\bar{\text{B}}{\left|\right|}_{\infty }+\gamma_{3}||\text{C}-\bar{\text{C}}{\left|\right|}_{\infty }+\left(\alpha_{1}+\alpha_{2}+\sigma 3\right)\left|\right|\bar{\text{R}}-\text{R}{\left|\right|}_{\infty }, \end{array}$$

$$\begin{array}{ccc} \left|\right|{\text{Z}}_{5}\left(\text{Y}\right)-{\text{Z}}_{5}\left(\bar{\text{Y}}\right){\left|\right|}_{\infty } & = & \left|\right|\phi_{3}\text{R}+\phi_{2}\text{C}+\phi_{1}\text{B}-\sigma_{3}\text{E}-\phi_{3}\text{R}-\phi_{2}\text{C}-\phi_{1}\text{B}+\sigma_{3}\text{E}\left|\right|\\ & \leq & \phi_{3}||\text{R}-\bar{\text{R}}{\left|\right|}_{\infty }+\phi_{2}||\text{C}-\bar{\text{C}}{\left|\right|}_{\infty }+\phi_{1}||\text{B}-\bar{\text{B}}{\left|\right|}_{\infty }+\sigma_{3}\left|\right|\bar{\text{E}}-\text{E}{\left|\right|}_{\infty }, \end{array}$$

*writing the above equation in matrix form, we get*:

$$\begin{array}{c} {}[\begin{array}{c} ||{\text{Z}}_{1}\left(\text{Y}\right)-{\text{Z}}_{1}\left(\bar{\text{Y}}\right)|{|}_{\infty }\\ ||{\text{Z}}_{2}\left(\text{Y}\right)-{\text{Z}}_{2}\left(\bar{\text{Y}}\right)|{|}_{\infty }\\ ||{\text{Z}}_{3}\left(\text{Y}\right)-{\text{Z}}_{3}\left(\bar{\text{Y}}\right)|{|}_{\infty }\\ ||{\text{Z}}_{4}\left(\text{Y}\right)-{\text{Z}}_{4}\left(\bar{\text{Y}}\right)|{|}_{\infty }\\ ||{\text{Z}}_{5}\left(\text{Y}\right)-{\text{Z}}_{5}\left(\bar{\text{Y}}\right)|{|}_{\infty } \end{array}]\leq \Theta [\begin{array}{c} ||\text{X}-\bar{\text{X}}|{|}_{\infty }\\ ||\text{B}-\bar{\text{B}}|{|}_{\infty }\\ ||\text{C}-\bar{\text{C}}|{|}_{\infty }\\ ||\text{R}-\bar{\text{R}}|{|}_{\infty }\\ ||\text{E}-\bar{\text{E}}|{|}_{\infty } \end{array}]. \end{array}$$ (21)

*where*

$$\begin{array}{c} \Theta =(\begin{array}{ccccc} \gamma_{1}+\mu_{1} & 0 & 0 & 0 & 0\\ \mu_{2} & \left(\gamma_{2}+\mu_{1}+\phi_{1}+\sigma_{1}\right) & 0 & \alpha_{1} & 0\\ 0 & \mu_{1} & \left(\gamma_{3}+\phi_{2}+\sigma_{2}\right) & \alpha_{2} & 0\\ \gamma_{1} & \gamma_{2} & \gamma_{3} & \left(\alpha_{1}+\alpha_{2}+\sigma_{3}\right) & 0\\ 0 & \phi_{1} & \phi_{2} & \phi_{3} & \sigma_{3} \end{array}). \end{array}$$ (22)

**Theorem 2** *Let us consider the matrix*

$$\begin{array}{c} \frac{\lambda^{1-\theta }\Psi_{\left(\theta \right)}^{\text{max}}\Phi_{\left(\theta \right)}^{\text{max}}}{\Gamma \left(\theta -1\right)|{\text{V}}_{0}\left(\theta \right)|}\Theta , \end{array}$$ (23)

*and it tends to* **O**_5_, *so system* 3 *and* 5 *will have a unique solution and the solution will be true for all* t>0 *in* $\bar{\text{U}}\left({\text{Y}}_{0},\Lambda \right)$.

**Proof 3.3** *Moreover, for any* **Y**, $\bar{\text{Y}}\in \bar{\text{U}}\left({\text{Y}}_{0},\Lambda \right)$, *further with the help of lemma 3.2, we have*:

||R(Y)-R(Y¯)||M=||λ1-θV0(θ)∫0texp(-V1(θ)V0(θ)(t-s))(D0RLt1−θZ(Y(s))-D0RLt1−θZ(Y¯(s)))||M≤λ1-θV0(θ)∫0t|exp(-V1(θ)V0(θ)(t-s))|||(D0RLt1−θZ(Y(s))-D0RLt1−θZ(Y¯(s)))||Mds≤λ1-θΨ(θ)maxΓ(θ-1)V0(θ)||∫0t(t-s)θ-2(Z(Y(s))-Z(Y¯(s)))ds||M≤λ1-θΨ(θ)maxΦ(θ)maxΓ(θ-1)V0(θ)||Z(Y)-Z(Y¯)||M≤λ1-θΨ(θ)maxΦ(θ)maxΓ(θ-1)V0(θ)Θ||Y-Y¯||M.

*Where* $\frac{\lambda^{1-\theta }\Psi_{\left(\theta \right)}^{\text{max}}\Phi_{\left(\theta \right)}^{\text{max}}}{\Gamma \left(\theta -1\right){\text{V}}_{0}\left(\theta \right)}\Theta$ *tends to 0, and the operator fulfills the criteria of* **G**-*contraction, so by using theorem 1 which is known as perov’s fixed point theorem, the systems* 3 *and* 5 *has only one solution lies in* $\bar{\text{U}}\left({\text{Y}}_{0},\Lambda \right)$. *Hence, the proof is complete*.

## 4 Existence and uniqueness of solution for our stochastic model 6

Let

$$\begin{array}{ccc} ð & = & (\text{X},\text{B},\text{C},\text{R},\text{E}),\\ ð(0) & = & (\text{X}(0),\text{B}(0),\text{C}(0),\text{R}(0),\text{E}(0)),\\ ð(t) & = & (\text{X}(t),\text{B}(t),\text{C}(t),\text{R}(t),\text{E}(t)),\\ \Xi & = & \text{X}+\text{B}+\text{C}+\text{R}+\text{E}. \end{array}$$

**Theorem 4.1** *We can find a non negative solution* (ð(t)) *for our proposed model with in the interval* 0≤t *along with initial values* $\left(ð\left(0\right)\right)\in R_{+}^{5}$ *and the solution will be in* $R_{+}^{5}$ *having probability one*.

**Proof 4.1** *If the coefficients of the model* (6) *fulfil the Lipschitz condition, a non-negative solution* (ð(t)) *be existent for the consider model over the time interval* $t\in [0,{\text{T}}_{e})$, *where* ${\text{T}}_{e}$ *denotes the time of occurrence. The initial value is given by* $\left(ð\left(0\right)\right)\in R_{+}^{5}$.

*In order to inaugurate the global nature of the solution for* (6), *it is essential to reveal that* **T**_*e*_ = ∞, *demonstrating an infinite time limit without an explosion*.

*Given* ${\bar{q}}_{0}$ *as a non-negative real number sufficiently large to guarantee that all initial values lie within the interval* $\left[\frac{1}{{\bar{q}}_{0}},\bar{q}\right]$ , *we can establish a stopping time for* $\bar{q}\geq {\bar{q}}_{0}$.

$$\begin{array}{ccc} {\text{T}}_{\bar{q}} & = & \left\{t\in \left[0,{\text{T}}_{e}\right):\;\text{min}\{ð(t)\}\leq \frac{1}{\bar{q}}\right\}\\ & or & \bar{q}\geq \text{max}\{ð(t)\}. \end{array}$$ (24)

*We assume that* inf(⌀) = ∞, *where* ⌀ *represents the empty set. The notation* ${\text{T}}_{\bar{q}}$ *is defined as increasing as* $\bar{q}\to \infty$. *Additionally, we suppose that* $\text{lim}\;\bar{q}\to \infty$ *so* ${\text{T}}_{\bar{q}}={\text{T}}_{\infty }$. *Our goal is to demonstrate that for all* t∈[0,∞), **T**_∞_ = ∞. *This indicates that* **T**_*e*_ = ∞, *and simultaneously confirms that* $(ð(t))\in {\text{R}}_{+}^{5}$. *If our supposition happens to be false, it implies the existence of a* **F** > 0 *and ξ* ∈ (0, 1) *so that*:

$$\begin{array}{c} \text{P}\{{\text{T}}_{\infty }\leq \text{F}\}>\xi . \end{array}$$ (25)

*Further, let’s take* $\text{X}:{\text{R}}_{+}^{5}\to {\text{R}}_{+}$ , *which is a function belonging to the* **C**^2^ *space, defined as*:

$$\begin{array}{c} \begin{array}{cc} \bar{\text{W}}(\Lambda )= & \bar{\text{W}}(ð)-\\ & 5-(\text{log}\;\text{X}+\text{log}\;\text{B}+\text{log}\;\text{C}+\text{log}\;\text{R}+\text{log}\;\text{E}). \end{array} \end{array}$$

*Utilizing the inequality* -1+y-logy≥0 *for all* y>0 , *we can demonstrate that* $\bar{\text{W}}\geq 0$. *Now, assuming* $\bar{q}\geq {\bar{q}}_{0}$ *and* **F** ≥ **F**_0_, *applying Ito’s results to* Eq (26), *we obtain*:

$$\begin{array}{c} \begin{array}{cc} d\bar{\text{W}}(ð) & =\text{L}\bar{\text{W}}(ð)dt+\mu_{1}\left(\text{X}-1\right)d\Pi_{1}\left(t\right)+\mu_{2}\left(\text{B}-1\right)d\Pi_{2}\left(t\right)\\ & +\mu_{3}\left(\text{C}-1\right)d\Pi_{3}\left(t\right)+\mu_{4}\left(\text{R}-1\right)d\Pi_{4}\left(t\right)++\mu_{5}\left(\text{E}-1\right)d\Pi_{5}\left(t\right). \end{array} \end{array}$$ (26)

*From the above equation, we obtain*:

$\text{L}\bar{\text{W}}:{\text{R}}_{+}^{5}\to {\text{R}}_{+}$ , *is represented by*:

$$\begin{array}{c} \begin{array}{cc} L\bar{W}(\Lambda ) & =(1-\frac{1}{\text{X}})(\omega_{1}-\left(\gamma_{1}+\mu_{2}\right)\text{X})+(1-\frac{1}{\text{B}})(\omega_{2}+\mu_{2}\text{X}+\alpha_{1}\text{R}-\left(\gamma_{2}+\mu_{1}+\phi_{1}+\sigma_{1}\right)\text{B})\\ & +(1-\frac{1}{\text{C}})(\omega_{3}+\mu_{1}\text{B}+\alpha_{2}\text{R}-\left(\gamma_{3}+\phi_{2}+\sigma_{2}\right)\text{C})+(1-\frac{1}{\text{R}})(\gamma_{1}\text{X}+\gamma_{2}\text{B}+\gamma_{3}\text{C}-\left(\alpha_{1}+\alpha_{2}+\phi_{3}\right)\text{R})\\ & +(1-\frac{1}{\text{E}})(\phi_{3}\text{R}+\phi_{2}\text{C}+\phi_{1}\text{B}-\sigma_{3}\text{E})+\frac{\xi_{1}^{2}+\xi_{2}^{2}+\xi_{3}^{2}+\xi_{4}^{2}+\xi_{5}^{2}}{2}\\ = & \omega_{1}-\left(\gamma_{1}+\mu_{2}\right)\text{X}-\frac{\omega_{1}}{\text{X}}+\left(\gamma_{1}+\mu_{2}\right)+\frac{\xi_{1}^{2}}{2}+\omega_{2}+\mu_{2}\text{X}+\alpha_{1}\text{R}-\left(\gamma_{2}+\mu_{1}+\phi_{1}+\sigma_{1}\right)\text{B}\\ - & \frac{\omega_{2}}{\text{B}}-\frac{\mu_{2}\text{X}}{\text{B}}-\frac{\alpha_{1}\text{R}}{\text{B}}+\left(\gamma_{2}+\mu_{1}+\phi_{1}+\sigma_{1}\right)+\frac{\xi_{2}^{2}}{2}+\omega_{3}+\mu_{1}\text{B}+\alpha_{2}\text{R}-\left(\gamma_{3}+\phi_{2}+\sigma_{2}\right)\text{C}\\ - & \frac{\omega_{3}}{\text{C}}-\frac{\mu_{1}\text{B}}{\text{C}}-\frac{\alpha_{2}\text{R}}{\text{C}}+\left(\gamma_{3}+\phi_{2}+\sigma_{2}\right)+\frac{\xi_{3}^{2}}{2}+\gamma_{1}\text{X}+\gamma_{2}\text{B}+\gamma_{3}\text{C}-\left(\alpha_{1}+\alpha_{2}+\phi_{3}\right)\text{R}\\ - & \frac{\gamma_{1}\text{X}}{\text{R}}-\frac{\gamma_{2}\text{B}}{\text{R}}-\frac{\gamma_{3}\text{C}}{\text{R}}+\left(\alpha_{1}+\alpha_{2}+\phi_{3}\right)+\frac{\xi_{4}^{2}}{2}+\phi_{3}\text{R}+\phi_{2}\text{C}+\phi_{1}\text{B}-\sigma_{3}\text{E}\\ - & \frac{\phi_{3}\text{R}}{\text{E}}-\frac{\phi_{2}\text{C}}{\text{E}}-\frac{\phi_{1}\text{B}}{\text{E}}+\sigma_{3}+\frac{\xi_{5}^{2}}{2}\\ \leq & \omega_{1}+\gamma_{1}+\mu_{2}+\omega_{2}+\gamma_{2}+\mu_{1}+\phi_{1}+\sigma_{1}+\omega_{3}+\gamma_{3}+\phi_{2}+\sigma_{2}+\alpha_{1}+\alpha_{2}+\phi_{3}+\sigma_{3}\\ + & \frac{\xi_{1}^{2}+\xi_{2}^{2}+\xi_{3}^{2}+\xi_{4}^{2}}{2}\\ \leq & \omega_{1}+\omega_{2}+\omega_{3}+\gamma_{1}+\gamma_{2}+\gamma_{3}+\mu_{1}+\mu_{2}+\phi_{1}+\phi_{2}+\phi_{3}+\sigma_{1}+\sigma_{2}+\sigma_{3}+\alpha_{1}+\alpha_{2}\\ + & \frac{\xi_{1}^{2}+\xi_{2}^{2}+\xi_{3}^{2}+\xi_{4}^{2}}{2}=\bar{\text{V}}. \end{array} \end{array}$$ (27)

*The significance of* $\bar{\text{V}}\geq 0$ *is that it remains constant, as it does not depend on any state or variable*.

$$\begin{array}{ccc} d\bar{\text{W}}(\Lambda ) & = & \bar{\text{V}}dt+\mu_{1}\left(\text{X}-1\right)d\Pi_{1}\left(t\right)+\mu_{2}\left(\text{B}-1\right)d\Pi_{2}\left(t\right)\\ & + & \mu_{3}\left(\text{C}-1\right)d\Pi_{3}\left(t\right)+\mu_{4}\left(\text{R}-1\right)d\Pi_{4}\left(t\right)+\mu_{5}\left(\text{E}-1\right)d\Pi_{5}\left(t\right). \end{array}$$ (28)

*Applying integration to the above* Eq (28), *we obtained*

$$\begin{array}{ccc} & & \bar{U}\left[\text{X}\left({\text{T}}_{\bar{q}}\wedge F\right),\text{B}\left({\text{T}}_{\bar{q}}\wedge \text{F}\right),\text{C}\left({\text{T}}_{\bar{q}}\wedge \text{F}\right),\text{R}\left({\text{T}}_{\bar{q}}\wedge \text{F}\right),\text{E}\left({\text{T}}_{\bar{q}}\wedge \text{F}\right)\right]\\ & \leq & \bar{\text{W}}(\text{X}\left(0\right),\text{B}\left(0\right),\text{C}\left(0\right),\text{R}\left(0\right),\text{E}\left(0\right)+\bar{U}[\int_{0}^{{\text{T}}_{\bar{q}}\wedge \text{F}}\bar{\text{V}}]\\ & \leq & \bar{\text{W}}\left({ð}_{0}\right)+\text{F}\bar{\text{V}}. \end{array}$$ (29)

*Let* $\Phi_{\bar{q}}$ *be well-defined as the set of all ϖ in* Φ *such that* $\text{F}\geq {\text{T}}_{\bar{q}}\left(\varpi \right)={\text{T}}_{\bar{q}}$. *This condition holds for* $\bar{q}\geq {\bar{q}}_{1}$. Eq 25 *can then be uttered as* $P(\Phi_{\bar{q}})\geq \xi$. *It can be shown that, for any specific value of ϖ, there exists at least one corresponding* $\text{X}({\text{T}}_{\bar{q}},\varpi )$, $\text{B}({\text{T}}_{\bar{q}},\varpi )$, $\text{C}({\text{T}}_{\bar{q}},\varpi )$, $\text{R}({\text{T}}_{\bar{q}},\varpi )$ *or* $\text{E}({\text{T}}_{\bar{q}},\varpi )$ *that is equal to either* $\bar{q}$ *or* $\frac{1}{\bar{q}}$.

$$\begin{array}{c} \bar{W}(\text{X}\left({\text{T}}_{\bar{q}},\varpi \right),\text{B}\left({\text{T}}_{\bar{q}},\varpi \right),\text{C}\left({\text{T}}_{\bar{q}},\varpi \right),\text{R}\left({\text{T}}_{\bar{q}},\varpi \right),\text{E}\left({\text{T}}_{\bar{q}},\varpi \right)\geq (\frac{1}{\bar{q}}-1-\text{log}\;\bar{q})\wedge \left(\bar{q}-1-\text{log}\;\bar{q}\right). \end{array}$$ (30)

*In the light of* Eqs (29) *and* (30), *we have*

$$\begin{array}{ccc} \bar{\text{W}}\left({ð}_{0}\right)+\text{F}\bar{\text{V}} & \geq & \bar{U}\left(I_{\Phi \bar{q}\left(\varpi \right)}\bar{\text{W}}\left(\text{X}\left({\text{T}}_{\bar{q}},\varpi \right),\text{B}\left({\text{T}}_{\bar{q}},\varpi \right),\text{C}\left({\text{T}}_{\bar{q}},\varpi \right),\text{R}\left({\text{T}}_{\bar{q}},\varpi \right),\text{E}\left({\text{T}}_{\bar{q}},\varpi \right)\right)\right)\\ & \geq & \xi [\left(\bar{q}-\text{log}\;\bar{q}-1\right)\wedge (\frac{1}{\bar{q}}-\text{log}\;\bar{q}-1)]. \end{array}$$

*The function* $I_{\Phi \bar{q}\left(\varpi \right)}$ *is certain about* Φ(*t*) *and functions as an indicator function. In the situation where* $\bar{q}$ *approaches infinity, then*

$$\begin{array}{c} \infty >\bar{\text{W}}\left({ð}_{0}\right)+\text{F}\bar{\text{V}}=\infty . \end{array}$$ (31)

*Here a contradiction arises, leading to* ${\text{T}}_{\bar{q}}=\infty$ , *the proof can be considered complete, given the context of the argument. This signifies a critical point where further logical progression may be impeded, leading to the fulfillment of the proof’s objectives*.

## 5 Numerical analysis

In this portion, we propose a numerical scheme based on CPC Grunwald-Letnikov nonstandard FDM. This scheme is for fractional and variable order, is given below:

(D0CPCtθx)(t)=νx(t),0<t≤T1,0<θ≤1,ν<0,x(0)=x0, (32)

(D0CPCtθ(t)x)(t)=νx(t),T1<t≤T2,0<θ(t)≤1,ν<0,x(T1)=x1, (33)

$$\begin{array}{ccc} x(t) & = & \nu x\left(t\right)+\varsigma x\left(t\right)dR^{{\text{H}}^{⋆}}\left(t\right),\;\;\;\;\;{\text{T}}_{1}<t\leq {\text{T}}_{f},\\ x({\text{T}}_{2}) & = & x_{2}, \end{array}$$ (34)

in the above equation R represent the Brownian motion and *ς* is the intensity while **H**^⋆^ represent Hurst index. The system 3 can be written as:

D0CPCtθx(t)=1Γ(1-θ)∫0t(t-s)-θ(V1(θ)x(s)+V0(θ)x′(s))ds=V1(θ)I0RLt1-θx(t)+V0(θ)D0Ctθx(t)=V1(θ)I0RLtθ-1x(t)+V0(θ)D0Ctθx(t), (35)

here, **V**_0_(*θ*) and **V**_1_(*θ*) both are kernels which is depending on *θ* also **V**_0_(*θ*) = *θ* **Q**^1−*θ*^, **V**_1_(*θ*) = (1 − *θ*)**Q**^*θ*^, where **Q** represent a constant, and *ϖ*_0_ = 1. Applying GLNFDM approach, 35 can be discretized as bellow:

D0CPCtθx(t)|t=tn=V1(θ)Π(Δt)θ-1(xn+1+∑i=1n+1ϖixn+1-i)+V0(θ)Π(Δt)θ(xn+1-∑i=1n+1κixn+1-i-rn+1x0),

where:

$$\begin{array}{c} \Pi \left(\Delta t\right)=\Delta t+\text{O}{\left(\Delta t\right)}^{2},\ldots ,0<\Pi \left(\Delta t\right)<1,\;\;\Delta t\to 0. \end{array}$$ (36)

Furthermore, Eq 32 can also be discretized as bellow:

$$\begin{array}{c} \frac{{\text{V}}_{1}\left(\theta \right)}{\Pi {\left(\Delta t\right)}^{\theta -1}}(x_{n+1}+\sum_{i=1}^{n+1}\varpi_{i}x_{n+1-i})+\frac{{\text{V}}_{0}\left(\theta \right)}{\Pi {\left(\Delta t\right)}^{\theta }}(x_{n+1}-\sum_{i=1}^{n+1}\kappa_{i}x_{n+1-i}-r_{n+1}x_{0})=\nu x\left(t_{n}\right), \end{array}$$ (37)

in the above expression, $\varpi_{0}=1,\;\;\varpi_{1}=\left(1-\frac{\theta }{i}\right)\varpi_{i-1},\;\;t^{n}=n\left(\Pi \left(\Delta t\right)\right),\;\;\Delta t=\frac{{\text{T}}_{f}}{{\text{N}}_{n}},\;\;{\text{N}}_{n}$ represent a natural number. $\kappa_{i}={\left(-1\right)}^{i-1}(\begin{array}{c} \theta \\ i \end{array})$, $\kappa_{1}=\theta ,\;\;r_{i}=\frac{i^{\theta }}{\Gamma (1-\theta )}$, where i=1,2,3,…n+1. Further, we extend our work according to the assumption given in [28]

$$\begin{array}{ccc} 0<\kappa_{i+1}<\kappa_{i}<\ldots <\kappa_{1} & = & \theta <1\\ 0<r_{i+1}<r_{i}<\ldots <r_{1} & = & \frac{1}{\Gamma (-\theta +1)}. \end{array}$$

So, if **V**_0_(*θ*) = 1 *and* **V**_1_(*θ*) = 0 in Eq 37 then one can easily do the discretization for Caputo operator using finite difference method.

Now discretizing Eq 33 we get:

$$\begin{array}{c} \frac{{\text{V}}_{1}\left(\theta \left(t_{i}\right)\right)}{{\left(\Pi \left(\Delta t\right)\right)}^{\theta (t_{i})-1}}(x_{n_{2}+1}+\sum_{i=n+1}^{n_{2}+1}\varpi_{i}x_{n_{2}+1-i})+\frac{{\text{V}}_{0}\left(\theta \left(t_{i}\right)\right)}{{\left(\Pi \left(\Delta t\right)\right)}^{\theta (t_{i})}}(x_{n_{2}+1}-\sum_{i=n+1}^{n_{2}+1}\kappa_{i}x_{n_{2}+1-i}-r_{n_{2}+1}x_{0})=\nu x\left({t_{n}}_{2}\right), \end{array}$$ (38)

here, $\varpi_{i}=1-\frac{\theta (t_{i})}{i}\varpi_{i-1},\;\;\kappa_{i}={\left(-1\right)}^{i-1}(\begin{array}{c} \theta (t_{i})\\ i \end{array}),\;\;‘\kappa_{1}=\theta \left(t^{i}\right),\;\;r_{i}=\frac{i^{\theta (t_{i})}}{\Gamma (1-\theta \left(t_{i}\right))}$. Moreover we discretized Eq 34 by using Euler-Maruyama method as given below:

$$\begin{array}{c} x\left(t_{n_{3}+1}\right)=x\left(t_{n_{3}}\right)+\nu x\left(t_{n_{3}}\right)\Pi \left(\Delta t\right)+\varsigma x\left(t_{n_{3}}\right)\Delta {\text{B}}_{n_{3}}+0.5x\left(t_{n_{3}}\right)\Pi {\left(\Delta t\right)}^{2{\text{H}}^{⋆}},\\ {\text{H}}^{⋆}>0.5,\;\;\;n_{3}=n_{2},\ldots ,k,\;\;\;\;\;{\text{T}}_{2}<t\leq {\text{T}}_{f}. \end{array}$$ (39)

## 6 Numerical illustrations

**Fig 1:**
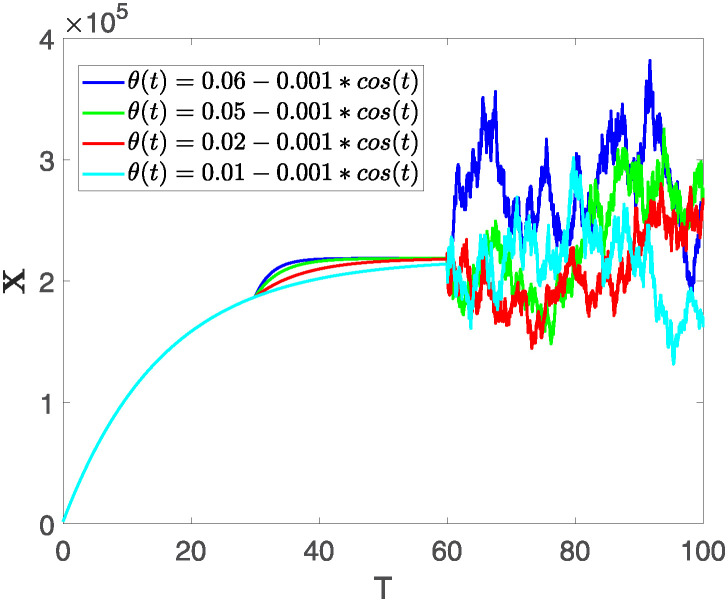
The population dynamics of state variable X of the system (6) with various values of *θ*(*t*), fractional order *θ* = 0.99 and parameters are used as *ς*_1_ = 0.1, *ς*_2_ = 0.1, *ς*_3_ = 0.1, *ς*_4_ = 0.1, *ς*_5_ = 0.1.

**Fig 2:**
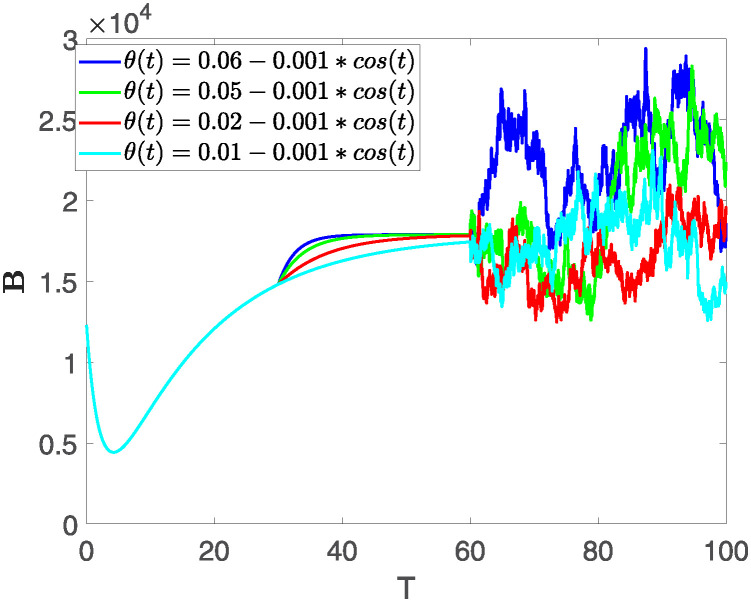
The population dynamics of state variable B of the system (6) with various values of *θ*(*t*), fractional order *θ* = 0.99 and parameters are used as *ς*_1_ = 0.1, *ς*_2_ = 0.1, *ς*_3_ = 0.1, *ς*_4_ = 0.1, *ς*_5_ = 0.1.

**Fig 3:**
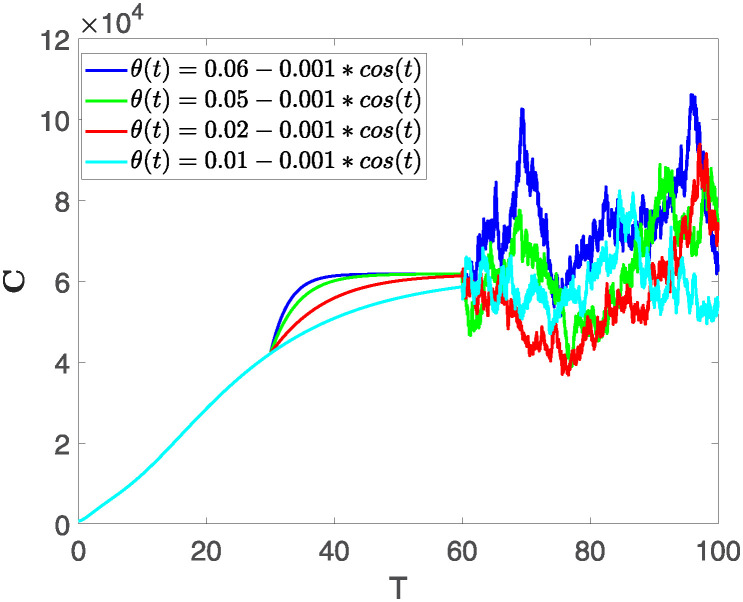
The population dynamics of state variable C of the system (6) with various values of *θ*(*t*), fractional order *θ* = 0.99 and parameters are used as *ς*_1_ = 0.1, *ς*_2_ = 0.1, *ς*_3_ = 0.1, *ς*_4_ = 0.1, *ς*_5_ = 0.1.

**Fig 4:**
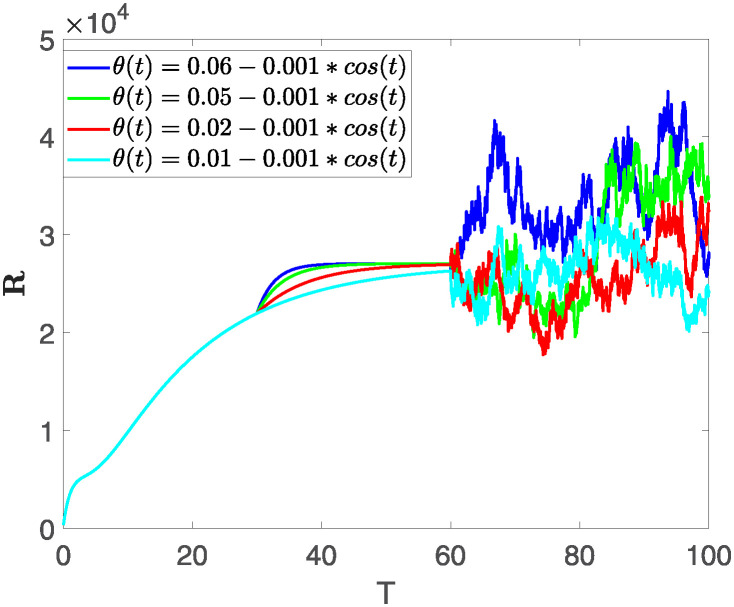
The population dynamics of state variable R of the system (6) with various values of *θ*(*t*), fractional order *θ* = 0.99 and parameters are used as *ς*_1_ = 0.1, *ς*_2_ = 0.1, *ς*_3_ = 0.1, *ς*_4_ = 0.1, *ς*_5_ = 0.1.

**Fig 5:**
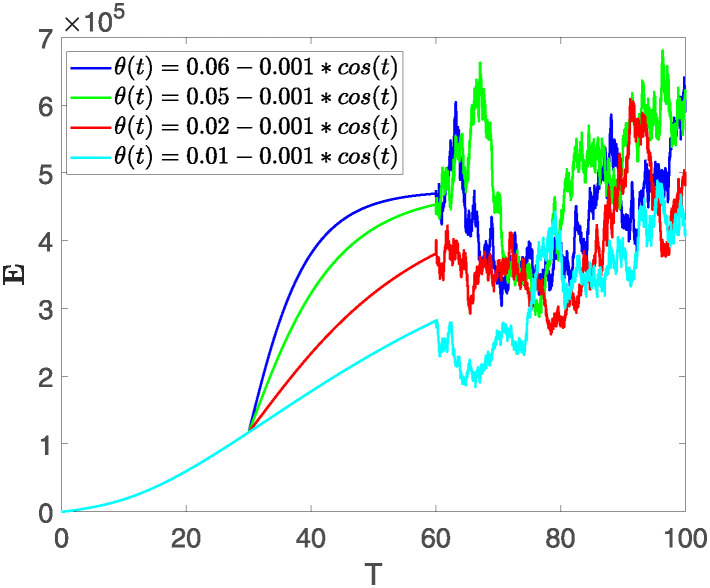
The population dynamics of state variable *I* of the system (6) with various values of *θ*(*t*), fractional order *θ* = 0.99 and parameters are used as *ς*_1_ = 0.1, *ς*_2_ = 0.1, *ς*_3_ = 0.1, *ς*_4_ = 0.1, *ς*_5_ = 0.1.

This section illustrates the visualization of the numerical findings using the proposed approach of the model outlined in Eq (6), showcasing a range of fractional orders alongside the modulation of key parameters. The time span is set from 0 to 100, with a step size of *dt* = 0.01. Here the interval is sub divided as [0, 30), [30, 60) and [60, 100], where the first one presents the fractional dynamics while the second interval presents the dynamics with variable order and the third interval presents the stochastic dynamics. The initial values are assumed to follow the pattern **X**(0) = 30000, **B**(0) = 12300, **C**(0) = 738, **R**(0) = 334, **E**(0) = 10. The parameters outlined in Table 1 are employed throughout the simulations. The behavior of state compartment **X** is visualized in Fig 1. It’s noticeable that the number of individuals admitted to hospitals and diagnosed with cancer is on the rise. The dynamics of state compartment **B**, is depicted in Fig 2. We notice in Fig 2, that in those people suffering from cancer with stages 3**X** and 3**B**, there’s a rapid initial decrease in their number of individuals, followed by a steady increase over time. Moreover, Figs 3 and 4 depict the evolution of individuals in classes **C** and **R**, respectively. From Fig 3, the trend shows a continuous rise in individuals with stage 4 cancer over time until *t* = 20, then we see that the number is large when *θ*(*t*) is large. Additionally, Fig 4 illustrates a growing number of individuals achieving disease-free status through chemotherapy as time progresses. In similar way, Fig 5 shows the dynamics of **E**, it’s evident that the population of cardiotoxic patients is steadily increasing over time which increases more rapidly in the second interval followed by stochastic dynamics.

**Table 1:** Parameters’ descriptions and their corresponding values.

Parameter	Description	Values	Source
Λ_1_	Patients diagnosed with cancer at stages 1 and 2	14000	[10]
Λ_2_	Individuals afflicted with cancer in stage 3	80	[10]
Λ_3_	Individuals afflicted with cancer in stage 4	80	[10]
*ϕ*_1_	For stages 1 and 2, the rate of recovery attributed to chemotherapy	0.03	[10]
*ϕ*_2_	For stage 3, the rate of recovery resulting from chemotherapy	0.4	[10]
*ϕ*_3_	For Stage 4, the rate of recovery stemming from chemotherapy	0.01	[10]
*β*_1_	The transmission rate of individuals progressing to stage 4 population	0.01	[10]
*β*_2_	The transmission rate of individuals to the class **B** population	0.01	[10]
*γ*_1_	The population rate experiencing cardiotoxicity induced by intensive chemotherapy	0.03	[10]
*γ*_2_	The population rate experiencing cardiotoxicity during stage 4 chemotherapy	0.03	[10]
*γ*_3_	The population rate experiencing cardiotoxicity due to intensive chemotherapy during the disease-free stage	0.03	[10]
*μ*_1_	Mortality rate at Stage 3 due to cancer	0.0256	[30]
*μ*_2_	Mortality rate at Stage 4 due to cancer	0.0256	[30]
*μ*_3_	The mortality rate of individuals experiencing cardiotoxicity	0.0256	[30]
*ψ*_1_	The recurrence of individuals reverting back to stage 3	0.03	[10]
*ψ*_2_	The regression of individuals back into stage 4	0.3	[10]

**Fig 6:**
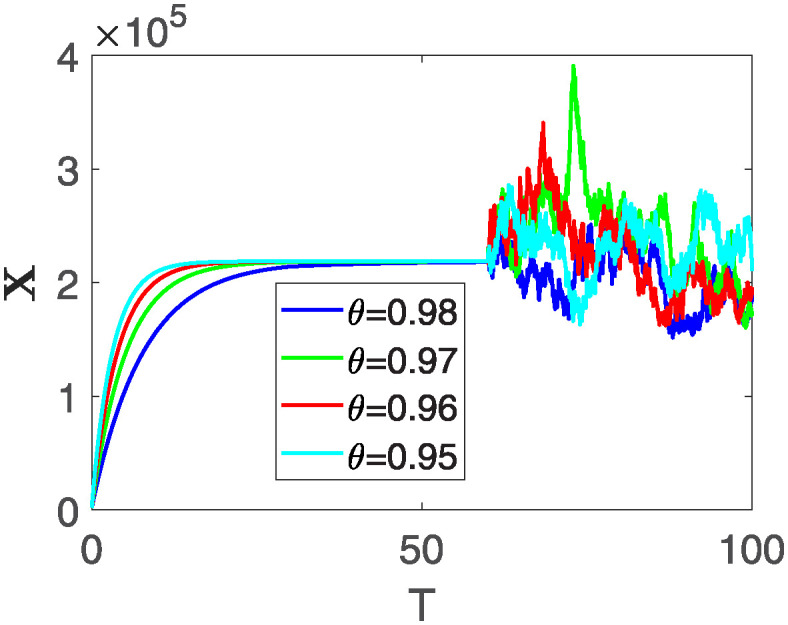
The population dynamics of state variable X of the system (6) with various values *θ*, variable order 0.01 − 0.001 * *cos*(*t*) and parameters are used as *ς*_1_ = 0.1, *ς*_2_ = 0.1, *ς*_3_ = 0.1, *ς*_4_ = 0.1, *ς*_5_ = 0.1.

**Fig 7:**
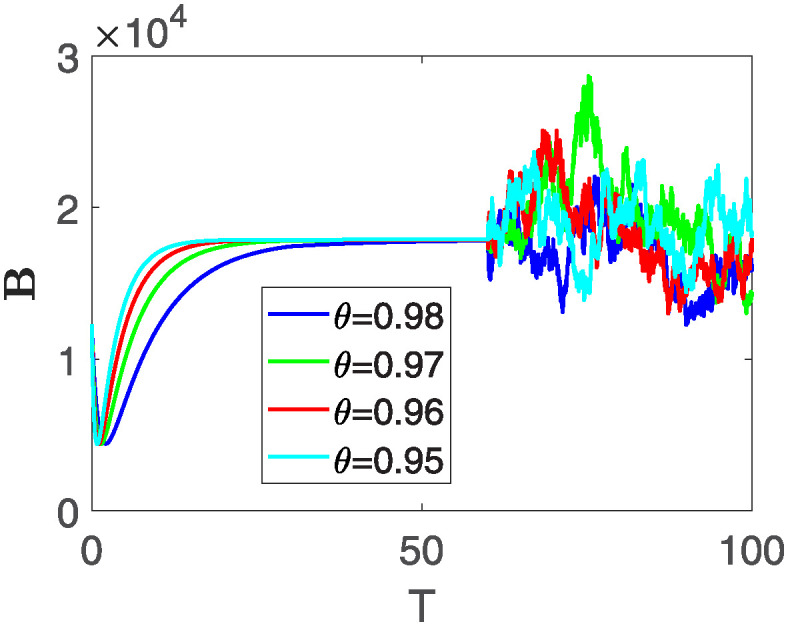
The population dynamics of state variable B of the system (6) with various values *θ*, variable order 0.01 − 0.001 * *cos*(*t*) and parameters are used as *ς*_1_ = 0.1, *ς*_2_ = 0.1, *ς*_3_ = 0.1, *ς*_4_ = 0.1, *ς*_5_ = 0.1.

**Fig 8:**
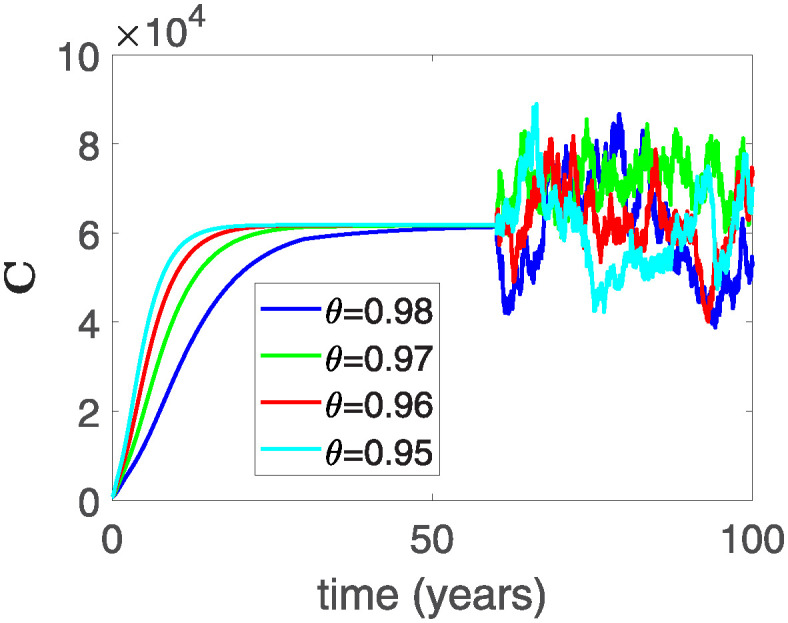
The population dynamics of state variable C of the system (6) with various values *θ*, variable order 0.01 − 0.001 * *cos*(*t*) and parameters are used as *ς*_1_ = 0.1, *ς*_2_ = 0.1, *ς*_3_ = 0.1, *ς*_4_ = 0.1, *ς*_5_ = 0.1.

**Fig 9:**
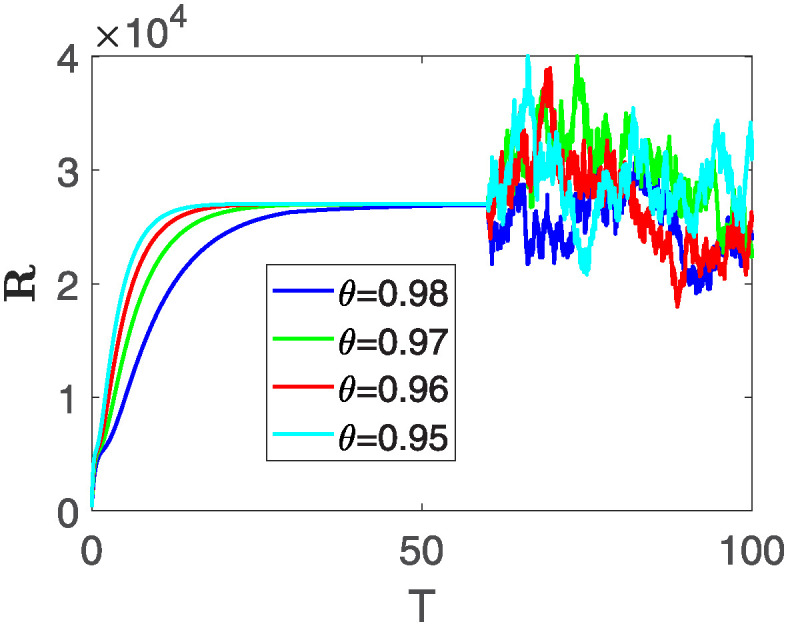
The population dynamics of state variable R of the system (6) with various values *θ*, variable order 0.01 − 0.001 * *cos*(*t*) and parameters are used as *ς*_1_ = 0.1, *ς*_2_ = 0.1, *ς*_3_ = 0.1, *ς*_4_ = 0.1, *ς*_5_ = 0.1.

**Fig 10:**
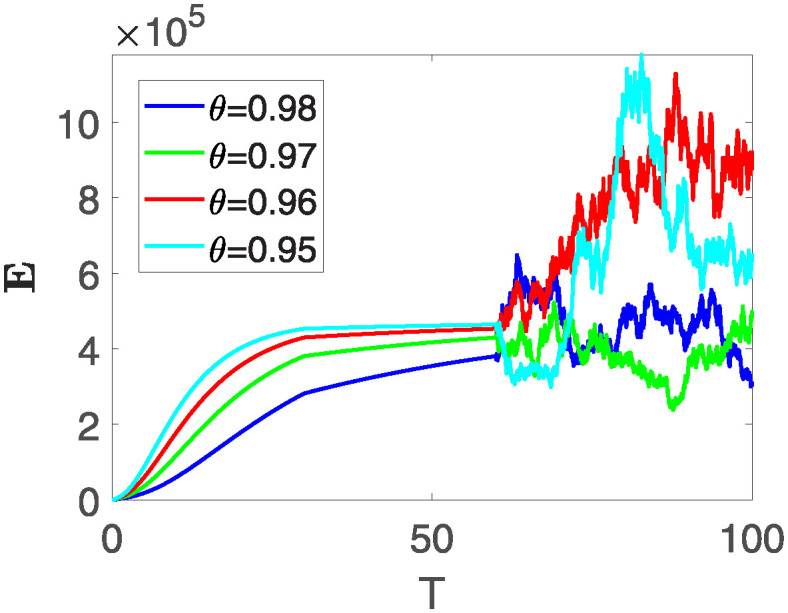
The population dynamics of state variable E of the system (6) with various values *θ*, variable order 0.01 − 0.001 * *cos*(*t*) and parameters are used as *ς*_1_ = 0.1, *ς*_2_ = 0.1, *ς*_3_ = 0.1, *ς*_4_ = 0.1, *ς*_5_ = 0.1.

Furthermore, the behavior of **X** is depicted in Fig 6 with the variations in fractional order *θ*. It’s noticeable that the number of individuals admitted to hospitals and diagnosed with cancer increases as fractional order decreases. The dynamics of **B**, is visualized in Fig 7. We see in Fig 7, that those people affected by cancer with stages 3**X** and 3**B**, there’s a rapid initial decrease in their number of individuals, then increase with time. Moreover, the Figs 8 and 9, depict the evolution of the individuals in **C** and **R**, respectively. From Fig 8, the trend shows a continuous rise in individuals with stage 4 cancer over time, then we see that there are more individuals when *θ* is small. Additionally, Fig 9 illustrates a growing number of individuals achieving disease-free status through chemotherapy as time progresses. In similar fashion, Fig 10 shows the dynamics of **E**, it’s evident that the population of cardiotoxic patients is steadily increasing over time which increases slightly faster in the second interval followed by stochastic dynamics.

**Fig 11:**
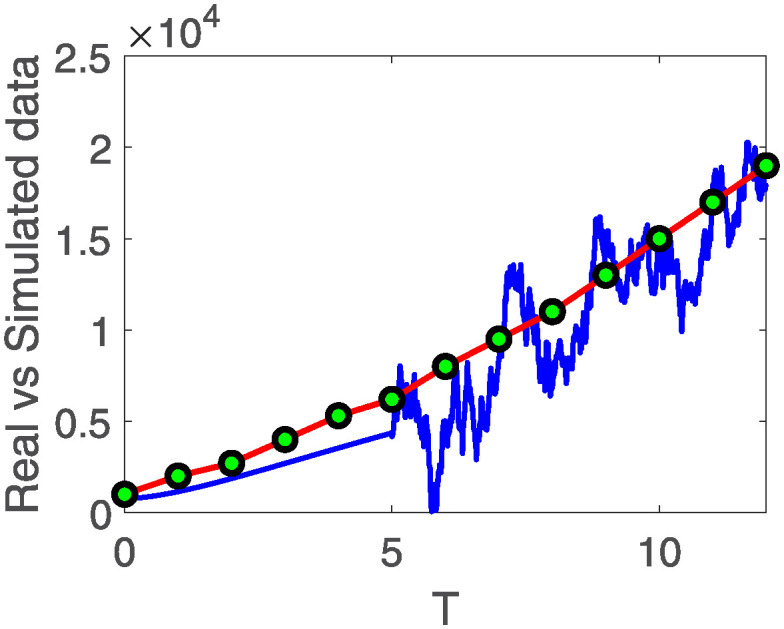
The system (6) with fractional order 0.9 evaluated by comparing real cases to simulated results spanning from 2004 to 2016 in the Kingdom of Saudi Arabia.

Fig 11 illustrates the comparison between real and simulated results, as examined in [31]. The time is considered to be [0, 12] with step size 0.01. The time interval is sub-divided as [0, 3), [3, 5) and [5, 12], where the first one presents the fractional dynamics with fractional order 0.9 while the second interval presents the behavior with variable order as variable order 0.01 − 0.001 * *cos*(*t*), and the third one shows stochastic dynamics. The data spans a period of 12 years, covering the years 2004 to 2016. The comparison highlights a significant overlap between the data points and the simulated results exhibiting stochastic behavior. This suggests that these operators possess the capability to provide improved predictions for cancer disease.

### 6.1 Parameters’ influence on the dynamics of the proposed model

**Fig 12:**
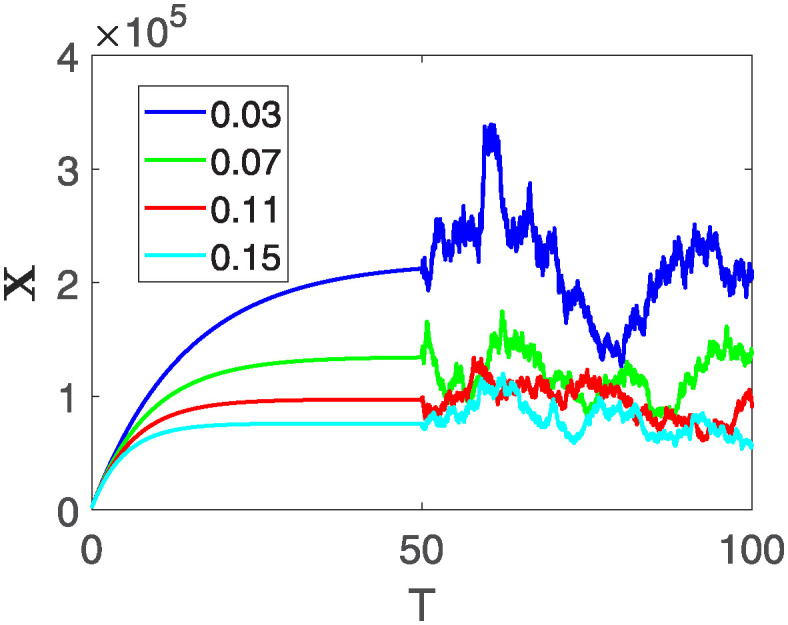
The population dynamics of the influence of *ϕ*_1_ on different state variable X of the system (6) with different values of *ϕ*_1_.

**Fig 13:**
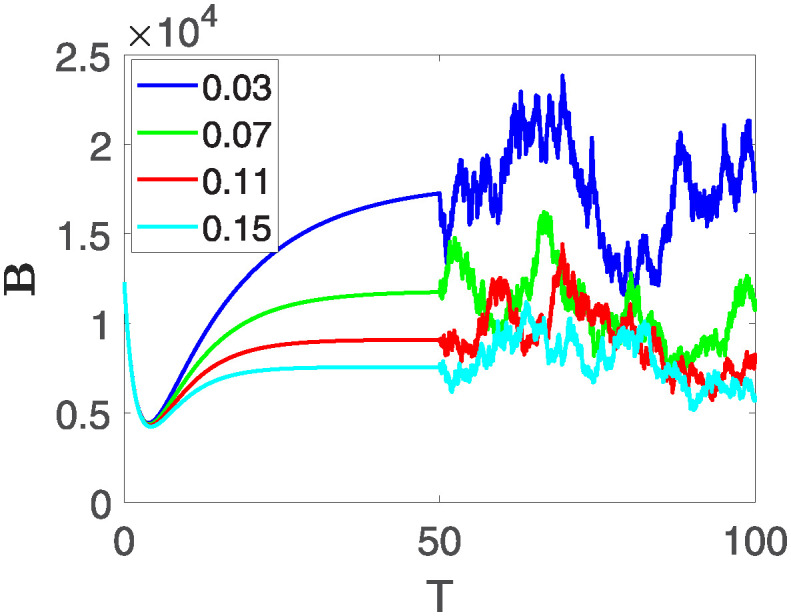
The population dynamics of the influence of *ϕ*_1_ on different state variable B of the system (6) with different values of *ϕ*_1_.

**Fig 14:**
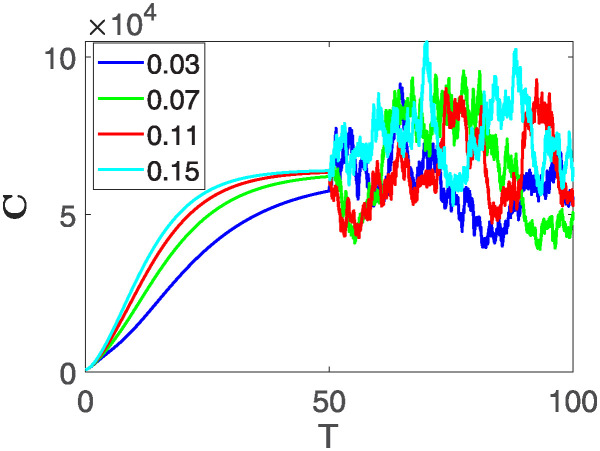
The population dynamics of the influence of *ϕ*_1_ on different state variable C of the system (6) with different values of *ϕ*_1_.

**Fig 15:**
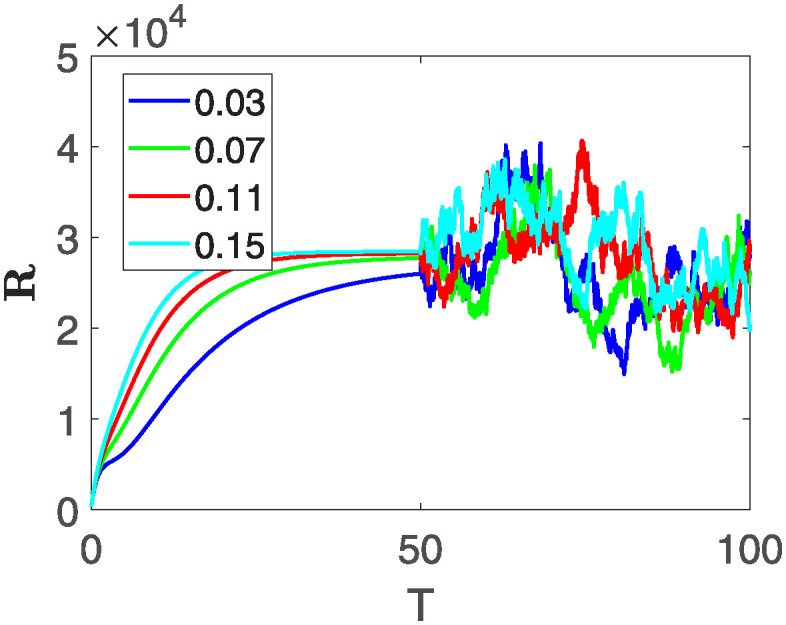
The population dynamics of the influence of *ϕ*_1_ on different state variable R of the system (6) with different values of *ϕ*_1_.

**Fig 16:**
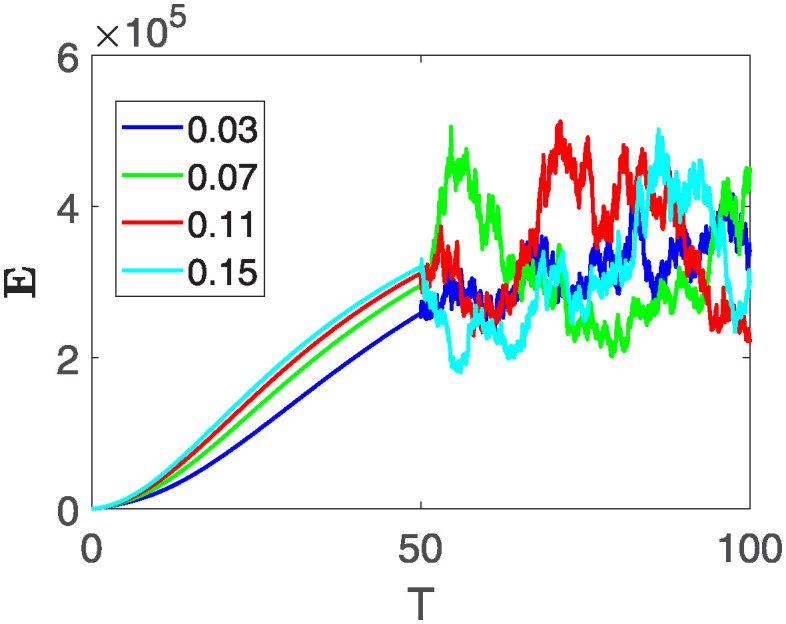
The population dynamics of the influence of *ϕ*_1_ on different state variable E of the system (6) with different values of *ϕ*_1_.

**Fig 17:**
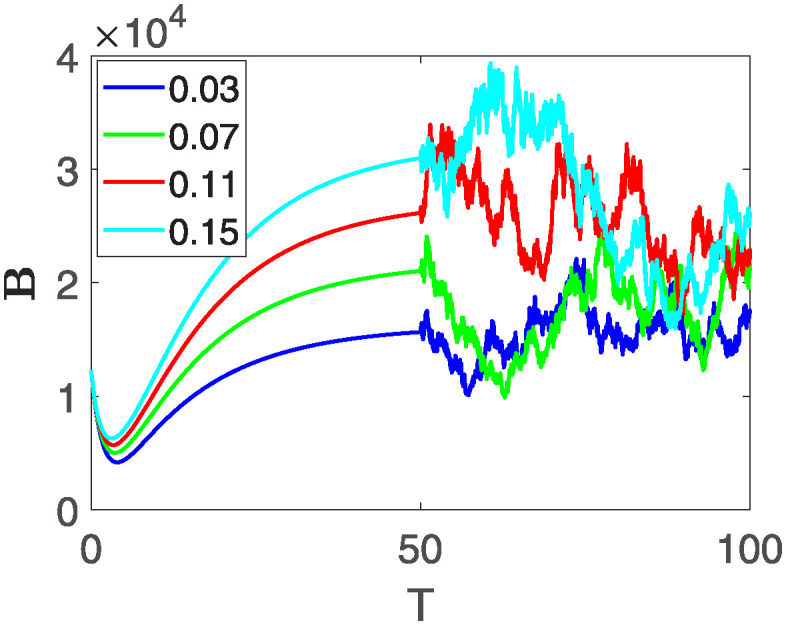
The population dynamics of different state variable B of the system (6) with different values of *ϕ*_3_.

**Fig 18:**
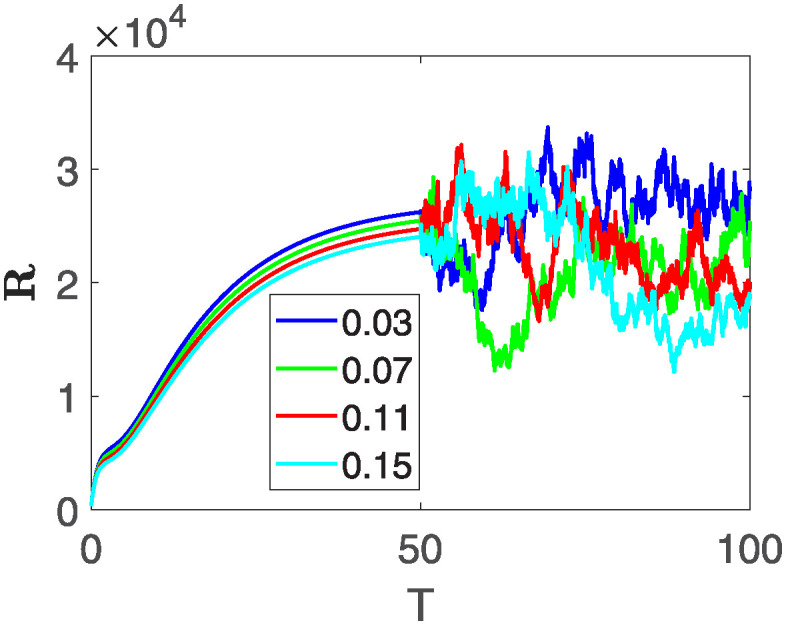
The population dynamics of different state variable R of the system (6) with different values of *ϕ*_3_.

**Fig 19:**
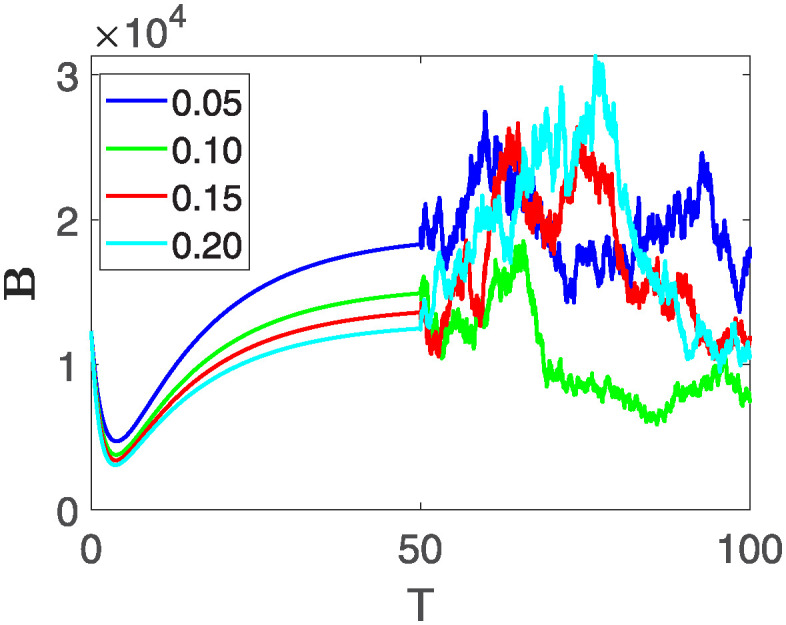
The population dynamics of different state variable B of the system (6) with various values of *γ*_1_.

**Fig 20:**
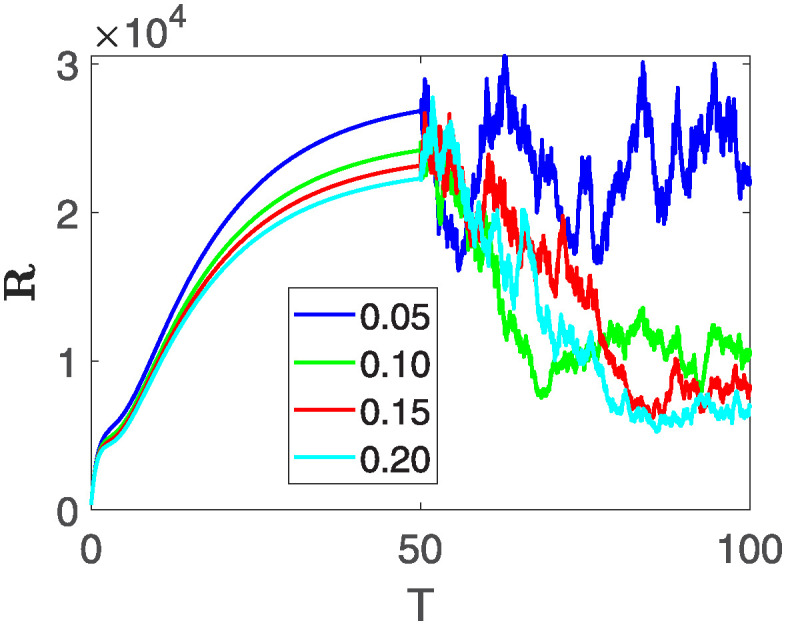
The population dynamics of different state variable R of the system (6) with various values of *γ*_1_.

**Fig 21:**
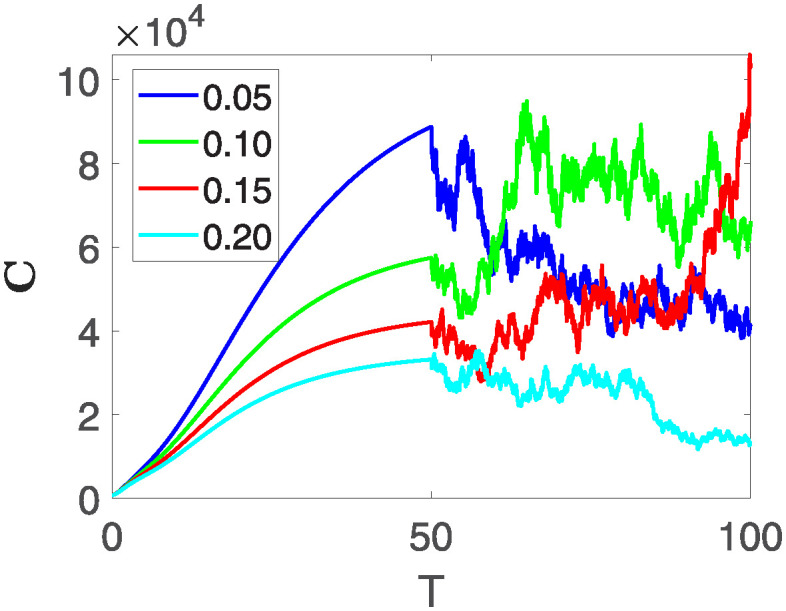
The population dynamics of different state variable C of the system (6) with various values of *γ*_2_.

**Fig 22:**
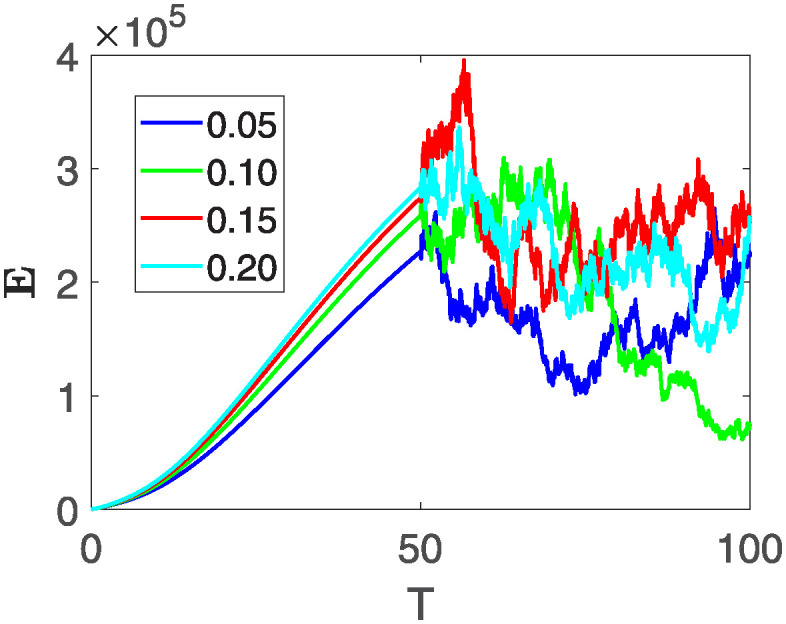
The population dynamics of different state variable E of the system (6) with various values of *γ*_2_.

This section of the current study is dedicated to examining the impact of various parameters on distinct state variables within the system. The objective is to ascertain whether augmenting specific parameters correlates with an increase in the population count within a particular class or if it yields an alternative effect. In the subsequent simulations, a fractional order of *θ* = 0.9 is utilized along with variable order as variable order 0.01 − 0.001 * *cos*(*t*). Henceforth, our initial focus revolves around exploring a range of values for the recovery rate during stages 1 and 2, attributed to chemotherapy, denoted as *ϕ*_1_. We examine values of 0.03, 0.06, 0.09, and 0.12 for *ϕ*_1_. From the figures, it is evident that as more individuals recover during stages 1 and 2, there is a notable decrease in the number of patients, highlighting the significance and efficacy of this outcome. Figs 12 and 13, demonstrate the number of people in **X** and **B** is decreased as the *ϕ*_1_ is increased. In same way, Figs 14–16, depicts that the individuals in the state variables I, F and E increases with increase in *ϕ*_1_. These figures illustrate that augmenting *ϕ*_1_ leads to an increase in individuals afflicted with stage 4, while concurrently resulting in a higher count of individuals recovering from the disease. Further, the impact of the rate of recovery at stage 4 with chemotherapy is investigated. For *ϕ*_3_, the values are utilized to be 0.03, 0.07, 0.11, 0.15. As depicted in Fig 17, a decrease in the number of individuals afflicted with stage 4 cancer is evident with an increase in chemotherapy at stage 4, particularly noticeable when the value of *ϕ*_3_ is elevated, as demonstrated in Fig 17. Furthermore, it has been observed that as chemotherapy is intensified at stage 4, there is a corresponding increase in the number of individuals who recover from the disease Fig 18. Now we show the dynamics of the influence of cardiotoxicity caused by intensive chemotherapy *γ*_1_ on **B** and **R**. From Fig 19, it can be seen that an increase in *γ*_1_ directly decreases the population in the presented state variables. The influence of increased cardiotoxicity in patients at stage 4 chemotherapy *γ*_2_, is demonstrated in Fig 20 with values considered as 0.05, 0.10, 0.15, 0.20. Figs 21 and 22 show that as *γ*_2_ increases, the number of individuals in **C** decreases, while the population and **E** increase, indicating that fewer people will recover from the disease. Cancer therapy has advanced significantly in recent years, substantially increasing the cure rate and preventing recurrences of breast cancer. However, the risk of cardiotoxicity reduces the usage of these medications. Cardiotoxicity, one of the most important side impacts of chemotherapy, substantially increases both mortality and morbidity rates, contributing to the slight increase observed in Fig 22.

## 7 Conclusion

Many operators have been defined in the literature to depict the dynamics of physical system. The PW operator which is helpful in the analysis of cross over dynamics of disease model, has been proposed very recently by Abdon and Seda. When the disease shows deterministic behaviour in one interval and stochastic dynamics in the other interval, the PW operator can be helpful for analysis of such types of behaviours. In this work, breast cancer, one of the most dangerous diseases affecting women, has been investigated using a piecewise differential operator. The considered operator has been divided into three sub-intervals. In the first sub-interval, a constant Caputo proportional operator (CPC) with a constant order has been applied. In the second sub-interval, the CPC operator has been used with a variable order. In the third sub-interval, a stochastic operator has been used to examine the stochastic dynamics of cancer cells. The study has had two primary focuses: theoretical and numerical analysis. For the theoretical analysis of the fractional order system with constant and variable fractional orders, fixed point theory has been employed to explore the existence and uniqueness of solutions in the first two sub-intervals. For the stochastic system, Ito calculus and nonlinear analysis have been used to demonstrate the existence of solutions in the third sub-interval. In terms of numerical analysis, the nonstandard finite difference technique has been applied to the fractional order models, and Euler-Maruyama method has been used for the stochastic system. Numerical simulations have been performed for the proposed operators, and the results have been compared with real data from Saudi Arabia covering the period from 2004 to 2016. Additionally, the effects of chemotherapy and cardiotoxicity on breast cancer cells in different scenarios are illustrated through graphs.

## Supporting information

S1 File The Supplementary data to this article is attached as a word file. (DOCX)
