# Supplementary material for: Fractional and stochastic modeling of breast cancer progression with real data validation
Source: PLoS One. 2025 Jan 10;20(1):e0313676. doi: 10.1371/journal.pone.0313676 (PMC11723547; doi:10.1371/journal.pone.0313676)
Supplement: S1 File — (DOCX) [file pone.0313676.s001.docx]

| **Table 1.** Distribution of breast cancer cases (𝑛 = 18,970) according to region, 2004–2016. | | | | | | | | | | | | | |
| --- | --- | --- | --- | --- | --- | --- | --- | --- | --- | --- | --- | --- | --- |
| Region Year incidence  n (ASR) | | | | | | | | | | | | | |
|  | 2004 | 2005 | 2006 | 2007 | 2008 | 2009 | 2010 | 2011 | 2012 | 2013 | 2014 | 2015 | 2016 |
| Asir | 27(5.7) | 40(8.1) | 48(9.4) | 38(7.6) | 49(9) | 46(9.1) | 69(12.2) | 70(11.6) | 62(10.8) | 82(13.1) | 88(11.6) | 99(12.8) | 103(12.6) |
| Baha | 15(11.3) | 10(8.5) | 7(5.2) | 12(8.7) | 13(10.8) | 12(10) | 23(17.5) | 32(20.8) | 25(15.7) | 24(15.5) | 18(9.1) | 20(10) | 15(7) |
| Jazan | 20(6.3) | 27(7.5) | 9(2.6) | 27(8) | 21(6) | 37(11.5) | 36(10.6) | 48(12.3) | 33(7.7) | 34(8.2) | 32(6.4) | 28(5.1) | 55(10.4) |
| Madinah | 36(10.6) | 71(11.3) | 66(16.6) | 67(15.9) | 56(12.2) | 68(16.1) | 85(21.3) | 85(19.1) | 112(24.8) | 97(20.2) | 85(15.9) | 82(15.2) | 92(15.9) |
| Hail | 14(9) | 15(9.3) | 27(16) | 24(12.5) | 22(11.9) | 20(11.4) | 26(14) | 29(14.5) | 34(15.5) | 51(23.2) | 34(14.4) | 55(20.5) | 46(19) |
| Qassim | 34(12.6) | 47(10.1) | 47(14.8) | 53(16.7) | 41(13.3) | 74(25) | 103(32.8) | 88(23.7) | 57(15.9) | 88(23.9) | 86(21.2) | 89(21.7) | 131(31.7) |
| Najran | 2(2.2) | 10(8.6) | 6(4.5) | 12(12.8) | 14(10.8) | 8(6.4) | 13(11.6) | 14(10.2) | 15(11.5) | 13(9.3) | 10(5.4) | 23(14.3) | 31(17.4) |
| Jouf | 16(17.5) | 15(16.9) | 10(9.8) | 13(11.1) | 21(19.4) | 24(22.5) | 19(19.8) | 25(20.8) | 34(31.6) | 25(20.3) | 30(21.3) | 26(19.5) | 31(25) |
| Tabok | 18(11.7) | 15(12.5) | 30(16.7) | 38(22.7) | 28(17.2) | 20(12.2) | 24(16.4) | 33(20) | 41(22.6) | 49(24.9) | 36(13.1) | 44(15.5) | 50(19.8) |
| Northern | 18(11.7) | 6(9) | 7(13.3) | 7(11.1) | 7(10.5) | 7(9.9) | 15(17.9) | 10(10.4) | 10(12.6) | 22(25) | 21(18.2) | 26(25) | 11(9.4) |
| Riyadh | 202(19.4) | 212(18.6) | 275(22.6) | 312(25.7) | 307(24.2) | 356(29.4) | 376(30.6) | 416(28.3) | 389(25.6) | 475(29.3) | 549(32.8) | 546(33) | 563(33.8) |
| Makkah | 227(19.1) | 249(20.9) | 240(18.2) | 375(28.8) | 298(21.6) | 360(26.4) | 338(24.2) | 379(23.6) | 396(23.3) | 449(25.3) | 412(21.7) | 493(26.4) | 578(29.7) |
| Eastern | 159(22.6) | 214(30) | 204(25.4) | 254(30.8) | 268(31.8) | 267(33.1) | 338(39.5) | 357(35.8) | 326(32.6) | 428(41) | 402(36.2) | 439(37.1) | 526(46.7) |


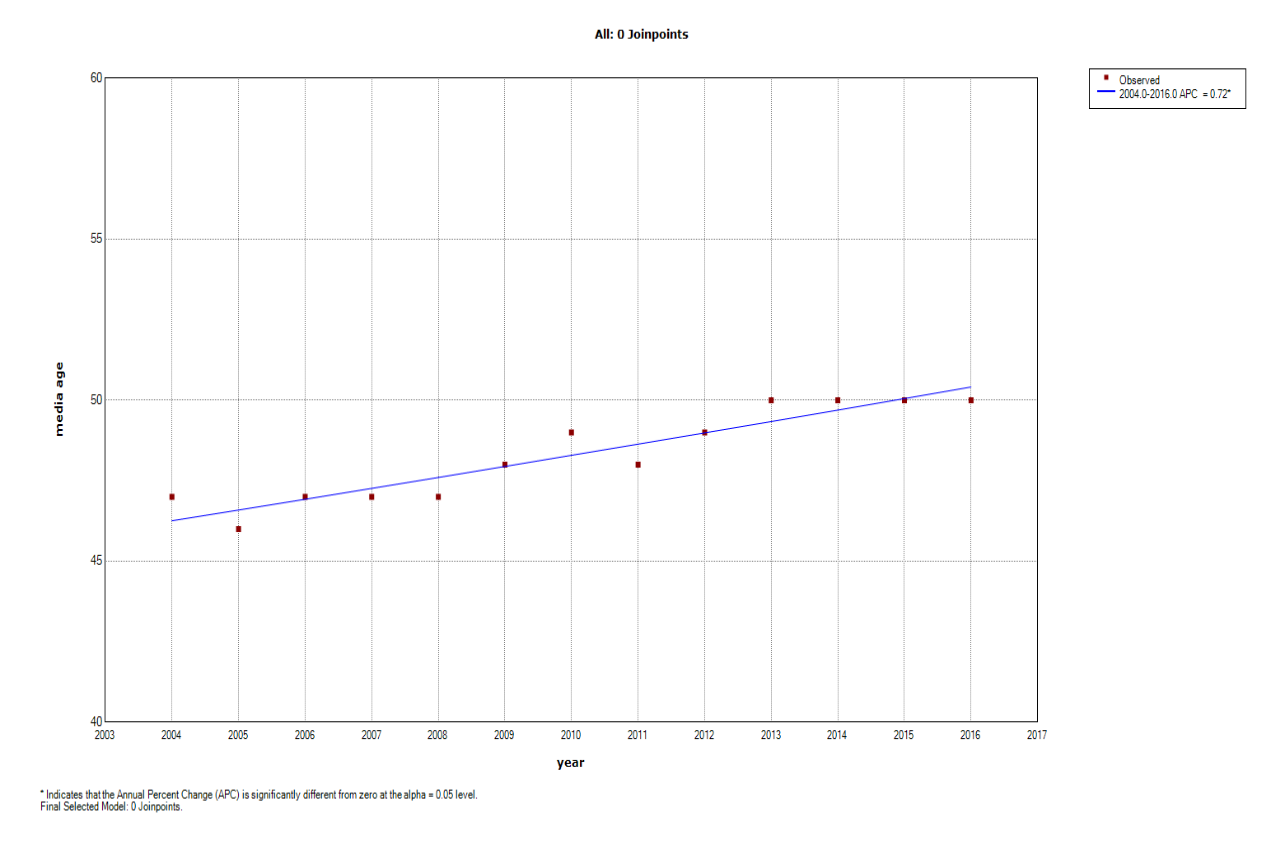


Figure 1. Joint point regression analysis of median ag at diagnosis of breast cancer in Saudi Arabia, 2004-2016.
